# Colossal Photovoltaic Current in Ferroelectric Oxide by Constructing Defect Band

**DOI:** 10.1002/advs.76000

**Published:** 2026-06-09

**Authors:** Yiran Sun, Zhenbang Dai, Xingzhong Cao, Peng Zhang, Chen Lin, Zihang Huang, Ruian Zhang, Yi Fu, Xuanyu Jiang, He Tian, Jingjing Xue, Kaifeng Wu, Junhui Wang, Tianqi Deng, Andrew M. Rappe, Gaorong Han, Zhaohui Ren

**Affiliations:** ^1^ State Key Laboratory of Silicon and Advanced Semiconductor Materials, School of Materials Science and Engineering Zhejiang University Hangzhou China; ^2^ Oden Institute For Computational Engineering and Sciences The University of Texas at Austin Austin Texas USA; ^3^ Department of Physics The University of Texas at Austin Austin Texas USA; ^4^ Institute of High Energy Physics Chinese Academy of Sciences Beijing China; ^5^ College of Materials Science and Engineering Zhejiang University of Technology Hangzhou China; ^6^ Institute of Advanced Semiconductors & Zhejiang Provincial Key Laboratory of Power Semiconductor Materials and Devices Hangzhou Global Scientific and Technological Innovation Center Zhejiang University Hangzhou China; ^7^ Center of Electron Microscope, School of Materials Science and Engineering Zhejiang University Hangzhou China; ^8^ State Key Laboratory of Molecular Reaction Dynamics and Dynamics Research Center for Energy and Environmental Materials Dalian Institute of Chemical Physics Chinese Academy of Sciences Dalian Liaoning China; ^9^ University of Chinese Academy of Sciences Beijing China; ^10^ Department of Chemistry University of Pennsylvania Philadelphia Pennsylvania USA; ^11^ Shanxi‐Zheda Institute of Advanced Materials and Chemical Engineering Taiyuan China

**Keywords:** defect band, ferroelectric photovoltaic, PbTiO_3_, Pb vacancies

## Abstract

Anomalous photovoltaic effect in ferroelectric materials has been intensively investigated in the past decades. However, an intrinsic current density in these materials remains very low at µA/cm^2^ level, hindering their device applications. Here, we report a colossal photovoltaic current density up to 34.36 mA/cm^2^ in PbTiO_3_ films with a Pb‐deficient layer under 375 nm light irradiation. The ultraviolet photoresponsivity at zero bias is 20 times higher than that of the emerged ferroelectric materials. A junction effect between the deficient and non‐defective layers in the film is proposed to produce the remarkable photovoltaic current density, while the photovoltage can be maintained by the anomalous photovoltaic effect. Notably, it was discovered that Pb vacancies introduce a defect band within the bandgap, enabling a direct transition from the valence band to the defect band. This transition accounts for a high‐efficient absorption and the prolonged carrier lifetime, thereby contributing to the superior photovoltaic performance. These findings suggest a new route to explore optoelectronic devices of ferroelectrics by the defect design.

## Graphical abstract

Colossal photovoltaic current density of 34.36 mA/cm^2^ under 375 nm illumination has been achieved in the Pb‐deficient PbTiO_3_ film. The ultraviolet photoresponsivity surpasses that of all reported ferroelectric materials. This enhancement is attributed to the junction effect between the Pb‐deficient and non‐defective layers. Importantly, Pb vacancies generate a defect band, which enables a direct transition from the valence band and thereby facilitates high‐efficient absorption and prolonged carrier lifetime.

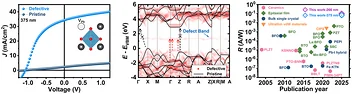

## 1 Introduction

The photovoltaic (PV) effect is at the center of optoelectronic applications from solar cells [1], photo‐electro‐catalysis [2] to optoelectronic detection [3]. In ferroelectric materials, PV effect was discovered about fifty years ago and exhibits unique characteristics, including above‐bandgap photovoltage [4], switchable PV current [5, 6], and inherent light polarization dependence [7, 8]. Different from the conventional junction PV effect, ferroelectric materials have a fundamentally different mechanism, based on polarization‐related charge‐separation. In particular, the above‐bandgap photovoltage in principle allows an energy conversion efficiency of ferroelectric materials higher than Shockley‐Queisser (S‐Q) limit in single‐junction device [9]. However, an intrinsic PV current in ferroelectric materials remains very low due to a combination of inefficient charge separation, substantial bulk recombination, and the critical influence of domain walls on carrier transport, severely limiting the practical application of ferroelectric materials in optoelectrical devices [10]. To enhance PV current, a widely adopted and effective approach is the band gap engineering. For instance, it has been demonstrated that two transition‐metal cations mixing of Ni^2+^ and Nb^5+^ in the solid state [KNbO_3_]_1−_ *_x_* [BaNi_1/2_Nb_1/2_O_3‐δ_]*_x_* were able to tailor the band gap and obtain a PV current of ≈4×10^−2^ µA/cm^2^ [7]. In PbTiO_3_ (PTO) system, the substitution of Ni is thought to work in conjunction with oxygen vacancies to alter the band structure and boost the PV current up to ≈10^−‍2^ mA/cm^2^ [11]. Recently, an innovative tactic has been proposed to improve PV current across a broad temperature by constructing superlattices involving BaTiO_3_ (BTO), SrTiO_3_ (STO), and CaTiO_3_ [12]. To realize an effective collection of carriers, a tip‐enhanced strategy has been well developed in single‐crystal BiFeO_3_ (BFO) [13] and BTO plates [14]. The aforementioned approach can even induce a bulk PV effect in STO, TiO_2_, and Si by breaking centrosymmetry via a strain gradient [15]. In addition, Bi‐site chemical substitution in BFO can destabilize the polar order, leading to an unstable polarization rotation that effectively inhibits the recombination of photoexcited carriers [16]. Nonetheless, despite great efforts in the past decades, the PV current density in ferroelectric materials is still at a very low level of µA/cm^2^.

One commonality of these emerging ferroelectric oxides is that almost all of them have an indirect gap, where a high‐efficiency absorption and subsequent direct transition are not allowed in theory, significantly suppressing large‐scale generation of photoexcited carriers. Moreover, an efficient separation of these carriers has not been realized in ferroelectric materials if a built‐in electric field is absence due to a complete screening. In contrast, such built‐in electric field is inherent to conventional p‐n junction devices. One can expect high photovoltage and large PV current if cooperative anomalous PV (APV) and junction PV effect is achieved in a single‐phase material. Here we realized a junction PV effect in ferroelectric PTO film with APV effect by introducing a Pb‐deficient layer. A colossal PV current of 0.27 mA (≈34.36 mA/cm^2^) and a photovoltage of 1.07 ‍V has been obtained in this film. The photovoltaic current was revealed to arise from the junction effect between Pb‐deficient and non‐defective layers, while the photovoltage is dominated by the APV effect. Interestingly, the existing Pb vacancies was proposed to generate a defect band, enabling an efficient optical absorption via a direct transition and supressing the electron‐phonon coupling near the valence band edge for a prolonged lifetime of carriers.

## 2 Results and Discussion

### 2.1 Construction of Pb‐Deficient Layer in Films

Point defects in ferroelectric oxide films have been widely explored to understand their effect on the spontaneous polarization and device performance [17, 18]. For example, oxygen vacancies in PTO were investigated to generally weaken ferroelectric polarization, while Pb vacancies appear to be benign [19]. In particular, oxygen vacancies can be the recombination center for the photoexcited electrons and holes [20], which is not helpful in separating the photocarriers and yielding desirable PV performance. In contrast, Pb vacancies can generate shallow defect states, which are shown in theory to be beneficial for the hole donor activity and the p‐type conduction in PTO [21]. Remarkably, Pb vacancies can be effectively stabilized by ferroelectric polarization even at high concentrations, which is challenging to achieve in other semiconducting material systems [22, 23]. It has been predicted that adjusting the chemical potential of oxygen can effectively enhance or diminish the concentration of Pb vacancies [24]. However, how to utilize the effective control over Pb vacancies to regulate PV effect of PTO remains elusive.

**FIGURE 1:**
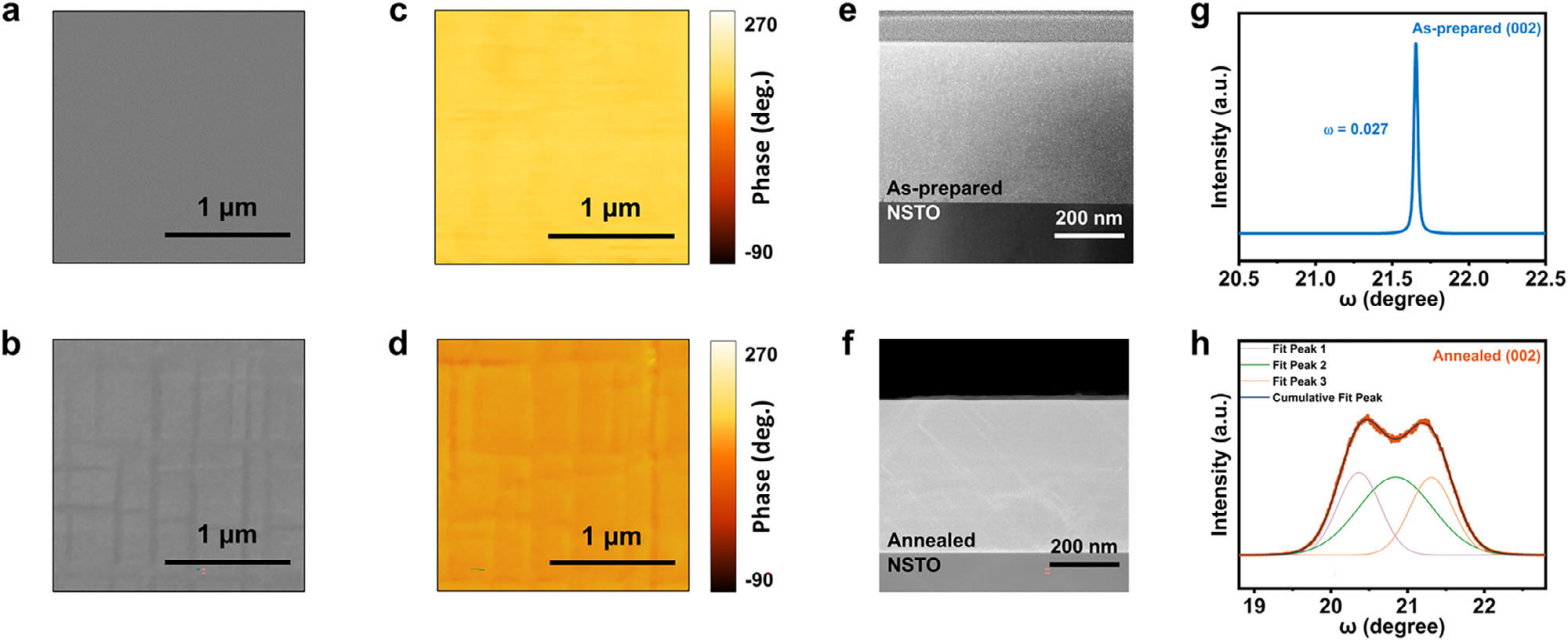
Morphological and structural characterization of as‐prepared and annealed PTO films. (a,b) SEM images of (a) as‐prepared and (b) annealed films. (c,d) Out‐of‐plane PFM phase images of (c) as‐prepared and (d) annealed films. (e,f) Cross‐sectional HAADF‐STEM images of (e) as‐prepared and (f) annealed films. (g,h) X‐ray rocking curves of (g) as‐prepared and (h) annealed films.

In this work, we constructed a Pb‐deficient layer of hydrothermal epitaxial PTO film [25] on 0.7 wt.% Nb doped SrTiO_3_ (NSTO) substrate by oxygen‐rich condition annealing. The films were annealed in an oxygen atmosphere for 2 h at different temperatures. The annealed film at 750°C was chosen for microstructure investigation. As shown in Figure 1a,b, both the as‐prepared and annealed films have smooth and flat surfaces. To further confirm the single‐crystal nature of the epitaxial PTO films, electron back‐scattered diffraction (EBSD) was performed on the as‐prepared film. A large‐area EBSD band contrast map (Figure S1a) and the uniform Z‐direction inverse pole figure (IPF Z, Figure S1b) demonstrate that the film exhibits single‐crystal characteristics with consistent crystallographic orientation. Due to the annealing temperature exceeding the Curie temperature (490°C), the domain structure of the films evolved from a single‐domain to many large‐scale 90° domains coexisting with very few 180° domains (Figure 1c,d; Figure S2). In addition, two films have a similar thickness of ≈445 nm (Figure 1e,f)). The epitaxial relationship of {001}_PTO_ || {100}_NSTO_ can be well identified by the XRD patterns of the tetragonal PTO (JCPDS 70–0746) film and cubic NSTO (JCPDS 35–0734) substrate (Figure S3). The single‐crystal characteristic of the annealed film is further supported by the off‐axis X‐ray reciprocal space mapping (RSM) in Figure S4b. The 103‐diffraction peak of film distinctly extends from 0.25 to 0.27 Å^−1^ in *Q*_x_ and from 0.71 to 0.74 Å^−1^ in *Q*_z_, respectively, reflecting a gradual change in the lattice spacing of (103) plane. Lattice parameter *c* gradually increases from 4.113 ± 0.011 Å (near the interface) to 4.175 ± 0.047 Å (near the surface), as the lattice parameter *a* decreases from 3.939 ± 0.013 Å to 3.920 ± 0.026 Å (Table S1). Compared with the as‐prepared film (Figure 1g), the splitting rocking curve of (002) for the annealed film (Figure 1h) can be explained by the existence of 90° domains [26]. A systematic comparison of films annealed at different temperatures (Figures S2 and S5) shows that the progressive splitting of the (002) rocking curve correlates directly with the gradual emergence of 90° domains observed by piezoelectric force microscope (PFM). The polarization direction can be determined to be mostly downward and in‐plane (Figure S6).

**FIGURE 2:**
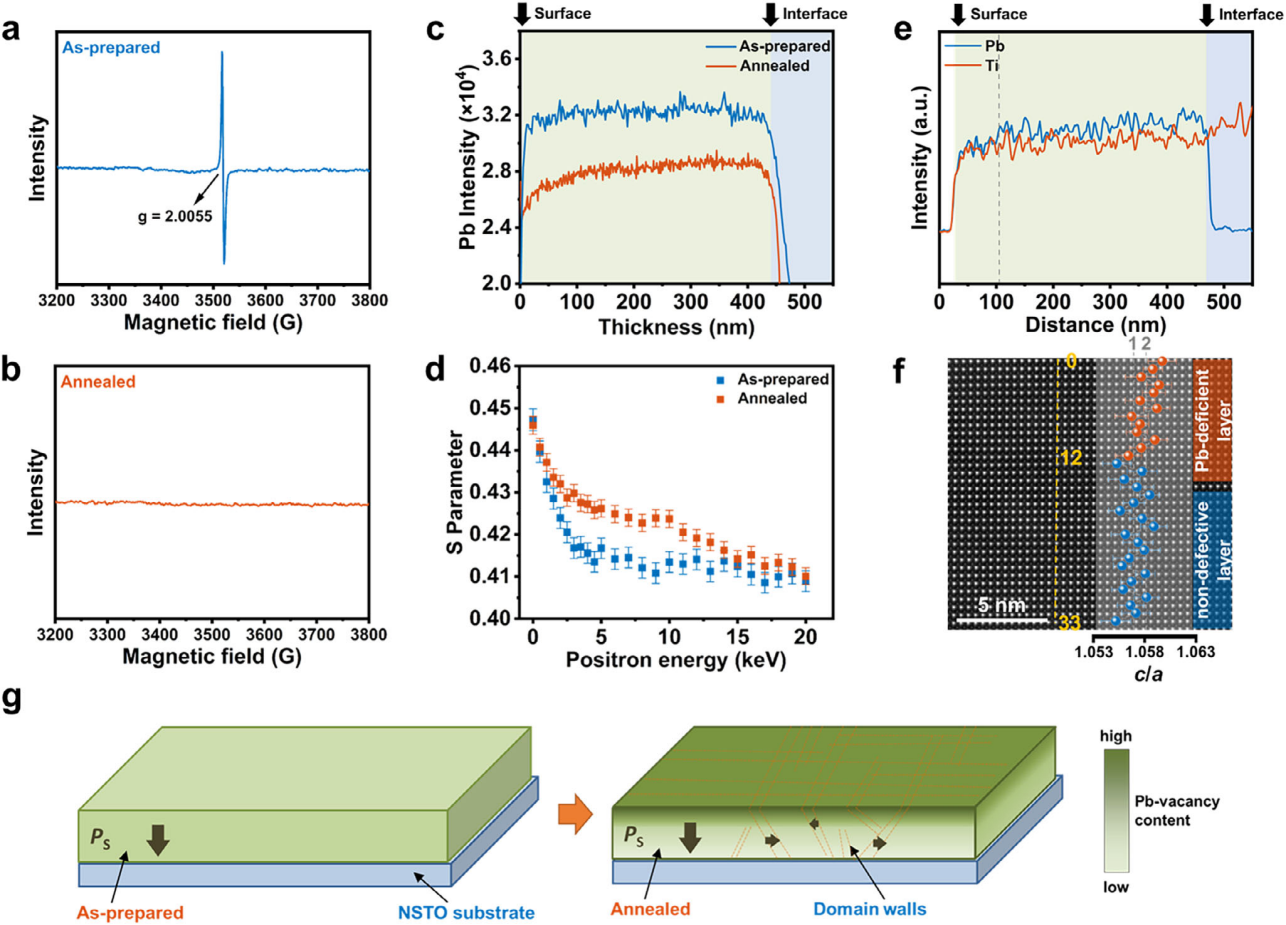
Characterization of point defects in as‐prepared and annealed PTO films. (a,b) EPR spectra of (a) as‐prepared and (b) annealed films. (c) TOF‐SIMS spectra of as‐prepared and annealed films for Pb element. (d) S‐parameter versus positron implantation energy of as‐prepared and annealed films. (e) STEM‐EDS line scan of the annealed film. (f) Atomic‐scale cross‐sectional HAADF‐STEM image of regions ≈100 nm from the surface. The inset is the lattice parameter ratio *c/a* averaged along atomic rows from top to bottom (corresponding to the vertical direction in (f)). The gray dashed lines 1 and 2 are the average values of the blue and orange atoms, respectively. (g) Schematic illustration of as‐prepared and annealed films. The color gradient in the surface ≈100 nm region of the annealed film schematically represents the Pb vacancy concentration, while the uniform color in the remaining film indicates a nearly constant and low Pb vacancy concentration.

In Figure 2a,b, the electron paramagnetic resonance (EPR) signal of oxygen vacancies of the annealed film almost disappeared, in stark contrast to that of the as‐prepared film, where the EPR signal of oxygen vacancies is located at g = 2.0055 [27]. This indicates a significant decrease in oxygen vacancies after the annealing process. Meanwhile, a distinct Pb gradient of films is investigated by time‐of‐flight secondary‐ion mass spectrometry (TOF‐SIMS) analysis, supporting a Pb‐deficient layer of the film. The concentration of Pb in the annealed film has a pronounced change, which decreases by ≈12.12% from the interface to the surface (Figure 2c). As a comparison, the Pb concentration in the pristine film changes slightly [25]. Positron annihilation spectroscopy (PAS) has been widely used for exploring charged defects [28]. As Figure 2d and Figure S7a show, it is evident that in the range of 0–15 keV positron energy, the S parameter for the annealed film is notably larger (while W parameter is smaller), implying a higher concentration of Pb vacancies within the PTO film. Moreover, S‐W curve (Figure S7b) exhibits an excellent linearity and a consistent slope, strongly supporting the existence of a single type of Pb vacancy. We also carried out HAADF‐STEM combined with energy‐dispersive X‐ray spectroscopy (EDS) line scan to analyze the elemental composition of the film (Figure 2e). Our results show that the Pb signal decreases from the surface to 100 nm deep into the film, suggesting a gradually decrease of Pb vacancies, matching well with the results of Figure 2c,d. Furthermore, we provided the atomic‐level HAADF‐STEM image of the annealed film at a position of ≈100 nm from the surface and statistically analyzed the variation in the *c*/*a* ratio in this region (Figure 2f). It was found that the presence of Pb vacancies leads to the deformation of the Ti‐O octahedra, which slightly increases the *c*/*a* ratio from 1.057 to 1.058. Based on the experimental results above, Pb vacancies were generated in the annealed film within the surface of ≈100 nm in thickness, as shown in the schematic (Figure 2g).

### 2.2 Photovoltaic Performance and Mechanism

**FIGURE 3:**
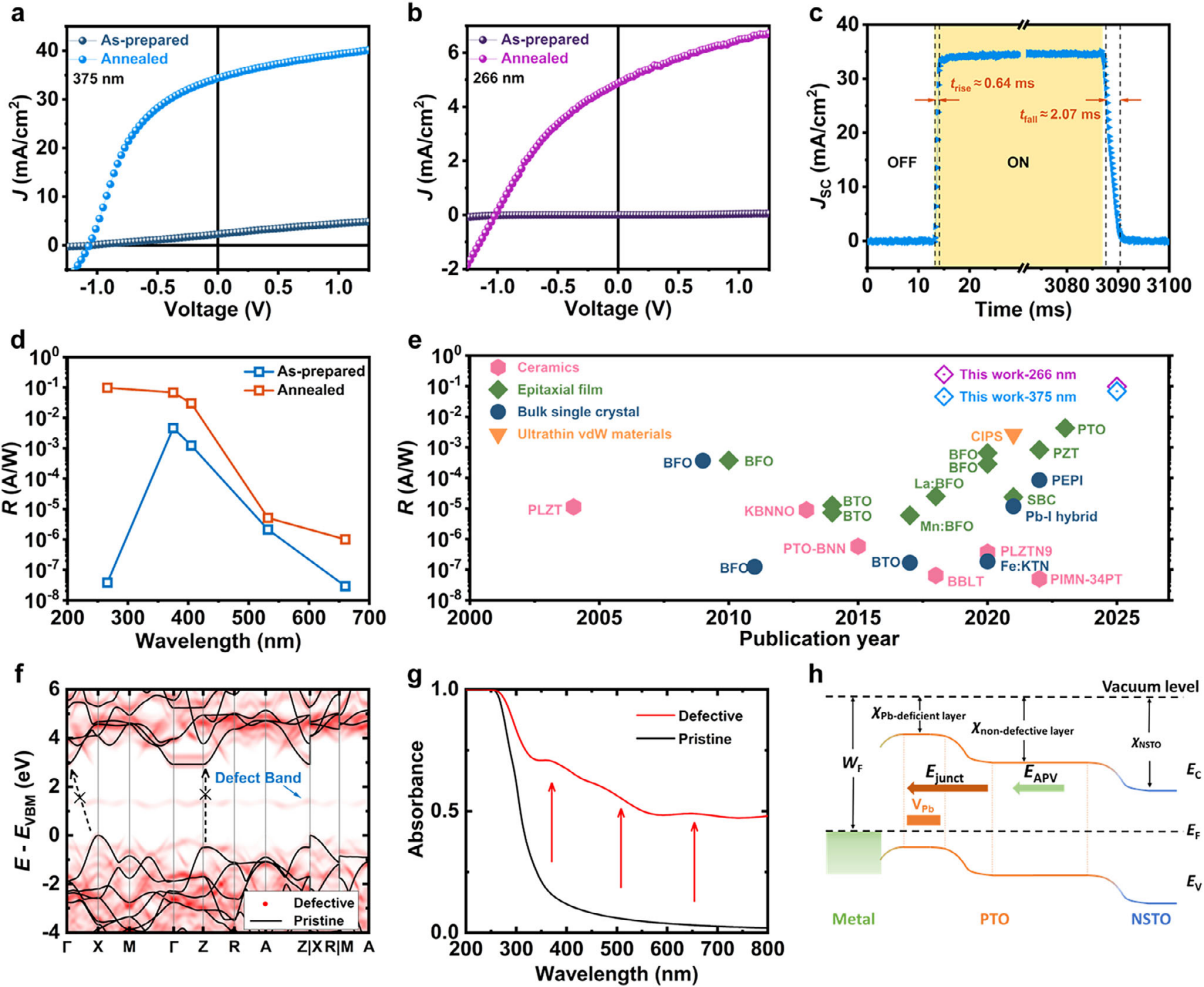
PV performance and theoretical mechanism of annealed PTO film. (a,b) *J–V* curves of as‐prepared and annealed films (a) under the illumination of 500 mW/cm^2^ light intensities (*I*_light_) from a 375 nm laser and (b) under the illumination of 50 mW/cm^2^ *I*_light_ from a 266 nm laser. (c) High‐resolution switch time response of annealed PTO film at zero bias under the illumination of 500 mW/cm^2^ *I*_light_ from a 375 nm laser. (d) Photoresponsivity‐wavelength curves of as‐prepared and annealed PTO films. The photoresponsivity values were derived from the short‐circuit current densities at the *I*_light_ specified in Table S2. (e) Photoresponsivity in annealed film (this work) compared with previously reported ferroelectric materials. (f) Electron band structure of defective PTO with 1/8 Pb vacancy (red) and pristine PTO (black). (g) Theoretical optical absorbance of defective PTO (red) and pristine PTO (black). The red arrows are guides to the eye showing enhanced absorption due to Pb vacancies. (h) Schematic of the energy band diagram of annealed film. *W*_F_, *χ*, *E*, and V_Pb_ represent work functions, electron affinity, built‐in electric field, and Pb vacancies, respectively.

The PV performance of the annealed films is investigated systematically (See detail experimental methods in Supporting Information). The PV current density‐voltage (*J*‐*V*) characteristics of the as‐prepared and annealed films under the illumination of 375 nm and 266 nm lasers are illustrated in Figure 3a,b, respectively. Under the excitation with a laser of 375 nm wavelength, the film exhibits a ≈14.9‐fold higher PV current density (*J*_SC_ ≈34.36 mA/cm^2^) than that of the as‐prepared one (≈2.31 mA/cm^2^). In particular, the PV response in deep UV band (*J*_SC_ ≈4.88 mA/cm^2^; 266 nm) has been achieved for the first time. Meanwhile, the open‐circuit voltage (*V*_OC_) of the film remains almost the same with small fluctuation from 1.02 to 1.07 V. More importantly, the *J‐V* curve transforms from a linear type to a nonlinear one, indicating different PV effects for the two films. The *J*_SC_‐*I*_light_ curves were fitted for both as‐prepared and annealed films in Figure S8c,d, where every *J*_SC_ value was extracted from Figure S8a,b at 100 s. For the as‐prepared film, the *J*_SC_ is linearly proportional to *I*_light_ with a power‐law fitting exponent of ≈0.98 (Figure S8c), well agreeing with APV effect that is generally described by the equation of *J*_SC_ *=* κ*·*α*·I*_light_. In this equation, κ is a constant depending only on the nature of the absorbing center and α is the absorption coefficient [29, 30]. In contrast, the *J*_SC_‐*I*_light_ curve is nonlinear (Figure S8d) for the annealed PTO film, where a power‐law exponent has been fitted to be 0.64 ($J_{\mathrm{SC}}\propto I_{\mathrm{light}}^{0.64}$). These results suggest that another possible PV effect could exist in the annealed film in addition to the APV effect. In addition, the response speed of the annealed film can be derived from the transition between ON and OFF states, as shown in Figure 3c. The rise time (from 10% to 90% of the maximum *J*_SC_ as switching light from OFF to ON) and the fall time (from 90% to 10% of the maximum *J*_SC_ as switching light from ON to OFF) of annealed film can be estimated to be 0.64 and 2.07 ms, respectively.

The annealed film also exhibits a pronounced photoresponsivity (*R* = *J*_SC_/*I*_light_) to the light from 266 nm to 660 nm wavelengths (Figure 3d; Table S2), due to the excitation from the valence band to the Pb vacancy level in the film [21, 24]. Obviously, the photoresponsivity of annealed film has improved from 0.00462 A/W to 0.0687 A/W. We also successfully prepared a ferroelectric film with an ultrahigh *R*, over 20 times as high as that of existing ferroelectric materials (Figure 3e; Table S3). Furthermore, we calculated the detectivity (D*) of PTO film to be 5.7 × 10^12^ Jones (Figure S9). In addition, different annealing temperatures allow to change surface Pb vacancies of the films, leading to different improvements of *J*_SC_ shown in Figures S10 and S11. The small diffraction peak adjacent to the substrate (002) peak in Figure S3 could be due to lead oxide‐related phase, which was revealed to make a negligible contribution to PV performance (Figure S12). Once the annealing temperature was above 800°C, the cracks were produced on the surface of the films, which would significantly undermine PV performance (Figure S13).

In order to understand the role of Pb vacancies, the film was polished to remove the Pb‐deficient layer (Figure S14a). It is interesting to find that the *J–V* curve of the sample becomes linear again, and *J*_SC_ performance is significantly decreased to ≈3.05 mA/cm^2^ (Figure S14b), which is close to that (≈2.31 mA/cm^2^) of as‐prepared film in Figure 3a. In particular, *V*_OC_ in Figure S14b is ≈1.02 V, similar to those of annealed (≈1.07 V) and as‐prepared (≈1.08 V) films. Furthermore, this nonlinear *J–V* curve can be well fitted by the following expression,

(1) $$J=a\cdot \exp\left(\frac{-V}{b}\right)+c+d\cdot V,$$

which corresponds to an effective circuit, involving a APV source is connected with a junction in parallel (Figure S15d). The physical background of the fitting formula is elaborated in Text SI. Thus, the fitting indicates a combined PV effect, which consists of junction‐like effect [31] and APV effect [25, 32, 33] and may lead to a colossal *J*_SC_ while maintaining *V*_OC_ of the system. The fitting results of data with 266 and 405 nm also agree with the expression (Figure S15). The homojunction is likely to form between the annealing generated Pb‐deficient layer and non‐defective layer, and one possible reason for the parallel connection is that as a result of the gradual change of the chemical composition of the Pb‐deficient layer, the junction region could be thick enough such that this region acts as both a junction with a barrier and a PV source. As the two effects happen at the same time and at the same location, then they may be understood as being connected in parallel. A homojunction here refers to an interface within the same PTO material, where the Pb‐deficient layer and the non‐defective layer form a built‐in electric field, while retaining an identical crystal structure. Moreover, we applied a dc (≈1.0 kV) bias to the Ag/PTO/NSTO devices in silicone oil for 3 min in order to switch their polarizations, and then investigated their PV performances. The direction of *J*_SC_ and *V*_OC_ in the as‐prepared film can be reversed by switching the polarization (Figure S16a), similar to the APV effect in solid state [KNbO_3_]_1−_ *_x_* [BaNi_1/2_Nb_1/2_O_3‐δ_]*_x_* (KBNNO) and CuInP_2_S_6_ (CIPS) films, which can be enhanced or suppressed subject to the electric polarization state [7, 34]. In contrast, the direction of *J*_SC_ and *V*_OC_ of the annealed films did not change after poling, and the *J*_SC_ only varied within the narrow range of 35.56–37.17 mA/cm^2^ (Figure S16b). Although the film can be switched, the reversal of polarization does not change the PV current significantly. We speculate that the polarization reversal slightly mediated the barrier height (Figure S16c,d) [35]. The conductive atomic force microscopy (c‐AFM) and surface potential measurements (Figures S17 and S18) on the annealed film indicate that the domain walls are not conductive and would not be beneficial for effective charge transport and aggregation near the surface under illuminated condition [36, 37]. Moreover, the domain walls exist in both annealed and polished films, while they demonstrate very distinct PV performance. Therefore, the domain walls make a negligible contribution to the enhancement of PV current in the annealed PTO film.

One should note that multiferroic Bi_2_FeCrO_6_ films have been demonstrated to generate a current of 11.7 mA/cm^2^ by using the Schottky effect [38]. To address this issue, we have prepared a series of as‐prepared PTO films with a thickness ranging from 120 ‍nm to 1.08 µm by adjusting hydrothermal reaction conditions and then investigated their PV performances (Figure S19a). It can be seen that *J*_SC_ and *V*_OC_ will first increase and then decrease as the film thickness grows, with the highest *J*_SC_ appearing in the film with a thickness of ≈445 nm. In addition, the PTO film with a thickness of ≈1.08 µm also exhibits a linear *J‐V* characteristic (Figure S19b). However, in theory the effective distance of the barrier at PTO/NSTO interface has been determined to be ≲50 nm for generating the PV current [35], which is at odds with the observation that the PV effect is strong throughout all film thicknesses under investigation, indicating that the Schottky barrier between the PTO film and the NSTO substrate cannot contribute to the observed PV performances. Furthermore, three top electrodes with different work functions (*W*_F_) have been deposited for PV measurements, including Ag (*W*_F, ‍Ag_ = 4.26 eV), Au (*W*_F, Au_ = 5.10 eV), and Pt (*W*_F, Pt_ = 5.65 eV). In theory, the larger *W*_F_ of the metal usually leads to the larger the net electric field and then the larger *J*_SC_. However, the results in Figure S20 can support a negligible contribution from the Schottky barrier since the electrode with largest *W*_F_ actually generates the smallest PV current. Hence, it can be concluded that the obtained PV performance is intrinsically derived from annealed PTO film.

We calculate the PTO band structure with 1/8 Pb vacancies and find that the generation of Pb vacancy forms an unoccupied defect band inside the original band gap (Figure 3f). This could introduce more holes due to the narrowed gap between valence band and unoccupied band. The presence of the defect band also significantly alters the optical absorption of the defective PTO. Specifically, the optical absorption from 300 to 700 nm are enhanced by orders of magnitude (Figure 3g). This is attributed to the transitions between the valence band and defect band, as well as the defect‐induced symmetry breaking, which allows some originally forbidden transitions in pristine PTO. Specifically, a high Pb vacancy density breaks the local symmetry of PTO, which allows some optical transitions that were previously forbidden by symmetry in pristine PTO. Subsequently, Pb vacancies introduce unoccupied in‐gap states as shown in Figure 3f. In this case, direct optical transitions with lower excitation energy become possible from the valence band at the Z/Γ point to the in‐gap defect states.

Due to a strong covalent bonding between the film and the substrate, it is very challenging to directly strip the film from NSTO substrate and separate contributions to optical absorption from the NSTO and the PTO film, which makes the interpretation of the experimental absorption spectrum elusive. To tackle this problem, we grew as‐prepared and annealed PTO films on a La_0.7_Sr_0.3_MnO_3_ substrate under similar conditions and stripped the PTO films by a method reported in Ref [39]. for measuring light absorption (Figure S21). We also made a detailed discussion in Text SII. The formation of Pb vacancies also induces a shift in electrostatic potential and, therefore, a built‐in electric field in the presence of vacancy concentration gradient (Figure S22). PFM measurements also indicate a larger built‐in electric field existing in the annealed film (Figure S23). Based on the calculation results, we propose the energy band diagram of annealed film (Figure 3h). The film retains the slight built‐in electric field as the APV material [25] in the pristine layer (As shown by the green arrow). Meanwhile, the two parts form a p+‐p homojunction due to the difference in Pb vacancy concentration, which generates the built‐in electric field to produce the giant PV current, and this diagram can also explain the nonlinear characteristics of the *J‐V* curve. Moreover, we qualitatively addressed this by examining films annealed at the same temperature (750°C) for different annealing time (0.5, 1, and 2 h). As annealing time increased from 0.5 h, 1 h to 2 h, the concentration of Pb vacancies became more pronounced, with the Pb‐deficient layer thickness increasing from ≈60 nm, ≈85 nm to ≈100 nm (Figure S24a). The increase in concentration of Pb vacancies leads to an increase in the number of carriers and the width of the space charge region within the annealed film, and simultaneously enhances the additional light absorption by defect states. Correspondingly, a substantial enhancement of *J*_SC_ has been increased from 5.99 mA/cm^2^, 19.13 mA/cm^2^ to 34.36 mA/cm^2^ (Figure S24b), supporting that Pb‐deficient absorption layer and the junction effect are critical for the resulting PV current of the films.

### 2.3 Dynamics of Carriers in Films

**FIGURE 4:**
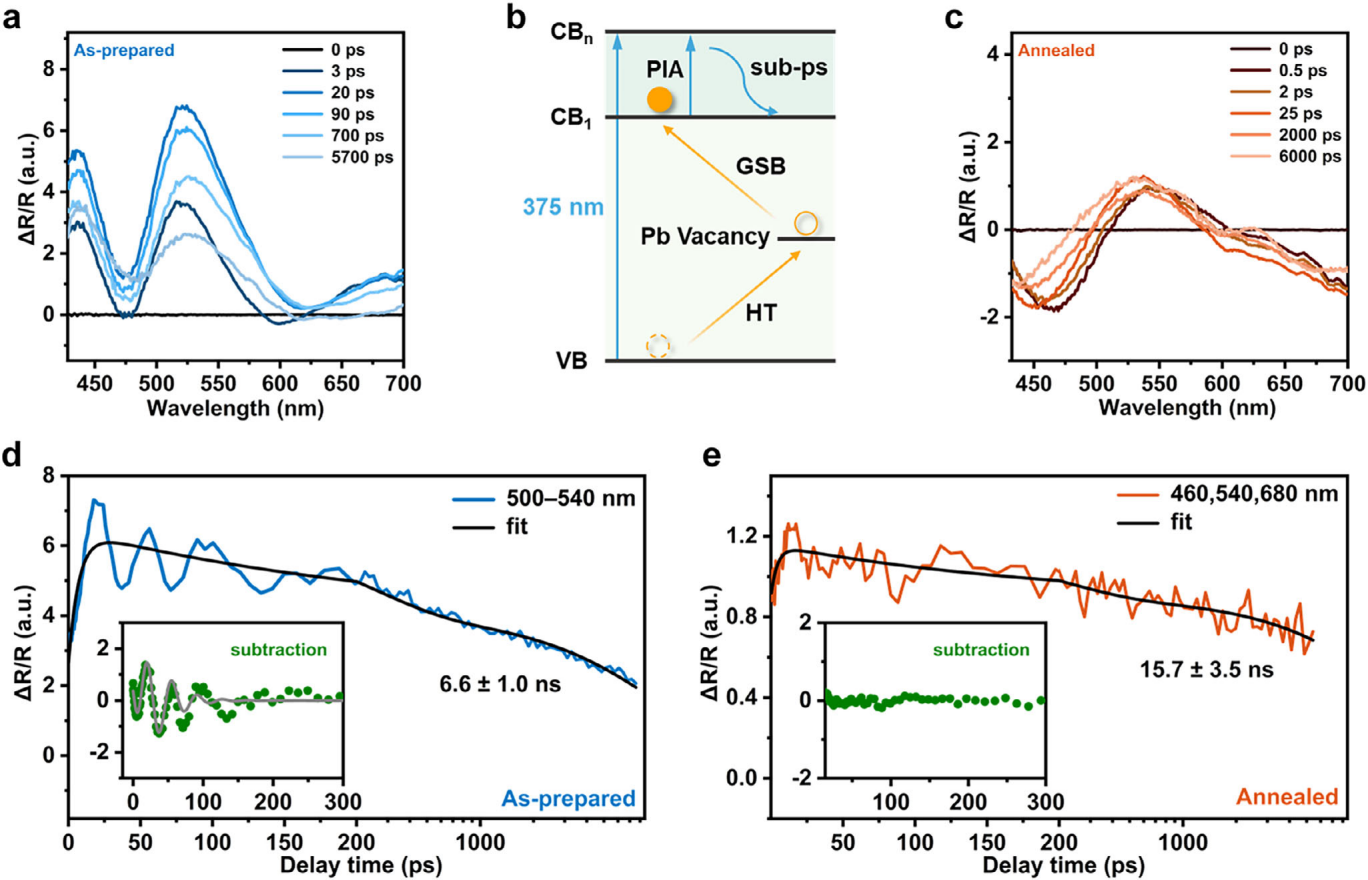
The TR spectra and kinetics of as‐prepared and annealed PTO films. (a) TR spectra at the indicated delays of as‐prepared films. (b) Schematic of energy level alignment and hole localization in the Pb vacancy in the PTO films. HT, PIA, and GSB represent hole transfer, photoinduced absorption, and ground state bleaches, respectively. (c) TR spectra at the indicated delays of annealed films. (d) TR kinetics probed at the range of 500–540 nm for as‐prepared film. The solid lines are biexponential fits to the data. The inset shows the enlarged phonon oscillation dynamics (green circles) due to the strong electron‐phonon coupling, obtained by taking the difference between the experimental TR kinetics (blue line) and the corresponding biexponential fitting data (black line), and its fits to damped sinusoidal functions (gray line). (e) TR kinetics for annealed film, obtained by averaging the decay curves at 460, 540, and 680 nm. The same subtracted kinetics are also shown in the inset for comparison.

To explore the electron kinetics after excitation, we performed ultrafast transient reflection (TR) [40, 41] measurement on the two PTO films under excitation at 375 nm with a fluence of 8.4 ‍mJ/cm^2^. As shown in Figure 4a,c, the TR spectra of both as‐prepared and annealed film at indicated delays exhibit a broad derivative‐like signal, with alternated photoinduced absorption (PIA, positive, 500–600 nm) and ground state bleaching signal (GSB, negative) peaked at ≈470 ‍nm. It should be noted that the GSB peak of the annealed film has an obvious blue shift with time evolution, as marked in Figure 4c. The spectral shift in perovskites and other low‐dimensional materials has been shown to originate from the built‐in electric field [42, 43], which motivated us to also correlate the spectral blue shift observed in our PTO films to the built‐in electric fields.

To clarify the physical processes underlying these spectral features, we schematically illustrate the excitation and charge transfer pathways in Figure 4b. In the ultrafast TR measurements, a pump beam at 375 nm was used to excite the excitonic transition from the valence band (VB) to the higher energy level of the conduction band (CB_n_) of the PTO films, generating hot electrons. The hot electrons in PTO films will rapidly relax to the bottom of the conduction band (CB_1_) on a sub‐ps timescale due to the efficient electron‐to‐hole energy transfer [44, 45, 46]. Therefore, all the following observed spectral evolutions originated from the electrons at the CB_1_. On the other hand, the existence of Pb vacancy provides an in‐gap level. This state is known to be occupied to reflect the Pb vacancy being negatively charged, so photogenerated holes in the VB tend to transfer to the Pb vacancy level in the film, as marked by the label HT in Figure 4b. It should be noted in the band structure (Figure 2f), the Pb vacancy level is unoccupied due to the setup of DFT calculations, where the Pb vacancy is constrained to be neutral. In more well‐refined calculations where different charge states were considered, it has been shown that the Pb vacancy under oxygen‐rich condition should be negatively charged [21]. Consequently, as shown in Figure 4a,c, the TR spectra characterized by PIA and GSB signals can be attributed to the transition from the CB_1_ to CB_n_ and from the Pb vacancy to CB_1_, respectively. More specifically, the photogenerated electrons and holes tend to transfer from CB_n_ to CB and from the VB to the Pb vacancy level in our PTO film following excitation. This spatial charge separate can be further facilitated by the built‐in electric field. Then, this spatially charge‐separated state, with electrons located at CB_1_ and holes localized at Pb vacancy, could dynamically screen the electron‐hole attraction, thereby reducing the exciton binding energy. The binding energy reduction would shift the exciton absorption toward a higher energy, leading to the spectral blue‐shift in our case [47]. As the exciton binding is reduced through this process, the photocarriers can be more effectively extracted, thus contributing to the PV performance enhancement in the annealed films. In addition, both of PIA and GSB signals reflect the dynamics of excited electrons and exhibit identical decay kinetics for PTO films, as indicated in Figure 4d,e. A separate fitting of the TR kinetics via a biexponential function

(2) $$I=A_{1}e^{-t/\tau_{1}}+A_{2}e^{-t/\tau_{2}}+I_{0},$$

where *A*_1_ and *A*_2_ are the amplitudes, *τ*_1_ and *τ*_2_ are the decay time constants and *I*_0_ is the baseline offset. This fitting yields the averaged electron lifetimes of 6.6 ± 1.0 ns and 15.7 ± 3.5 ns for the as‐prepared and annealed film, respectively, revealing an enhancement of the carrier lifetime by a factor of 2.4 in the annealed film. Such enhancement can be attributed to the more efficient carrier separation by the stronger built‐in electric field in the surface‐bulk junction.

Another reason of the prolonged lifetime may be the suppressed electron‐phonon coupling in the Pb‐deficient surface. To see this, it is interesting to note that, different from the annealed film (Figure 4e), the TR kinetics in the as‐prepared film clearly exhibits the coherent oscillation signal (Figure 4d), which may be attributed to the strong electron‐phonon coupling. By taking the difference between the experimental TR kinetics and the corresponding biexponential fitting curve, we can remove the electron signal background and isolate the pure oscillation dynamics for phonon modes, as shown in the inset of Figure 4d. This kinetic trace can be fitted to damped sinusoidal functions:

(3) $$S\left(t\right)\propto e^{-t/T}\;\mathrm{Sin}\left(\omega_{L}t+\varphi \right),$$

where *T* is the dephasing time, *ω*_L_ is the phonon oscillation frequency, and *φ* is the initial phase. The fitted phonon energy of as‐prepared film is 0.13 meV, which corresponds to the frequency of typical acoustic phonons (Figure S25). With this information, we calculated deformation potentials of the perfect PTO and Pb‐deficient PTO (Table S4). The results indicate that the interactions between electrons in the conduction band and the acoustic phonons are significantly reduced in the presence of Pb vacancies. Therefore, the formation of Pb vacancies in the annealed film will induce a reduction of the electron‐phonon coupling, which acts as another factor for the carrier lifetime enhancement, as also observed in metal halide perovskites [48].

Based on the results above and in the last section, we believe that the colossal PV current could be attributed to the enhanced optical absorption, the built‐in electric field, and the prolonged carrier lifetime. We also presented a drift‐current model to quantify the contribution from these three factors in Text SIII.

## 3 Conclusions

In summary, a combination of junction and APV effect has been realized in ferroelectric PTO films via construction of a Pb‐deficient layer. A junction effect between the deficient and non‐defective layers in the film is proposed to produce the colossal PV current (≈34.36 mA/cm^2^) and the APV effect mainly contributes to the photovoltage of ≈1.07 V. Such PV effect even allows the photoresponsivity at 266 nm and zero bias to exceed those of traditional semiconductor detectors, such as silicon, gallium oxide and gallium nitride based p‐n or Schottky junction [49, 50, 51]. In‐depth understanding of the critical role of Pb vacancies in optical absorption, electron‐phonon coupling, carrier dynamics provides new insights into regulating PV performances in perovskite ferroelectric oxide device and hybrid perovskite solar cells [52]. For more general implications, high‐concentration photogenerated carriers coupling with polarization, piezoelectricity or magnetism may leave us a wide field to explore interesting physics in ferroelectrics and multiferroics [10].

## 4 Experimental Section

Details of the experiments and methods are provided in the Supporting Information.

## Author Contributions

**Zhaohui Ren** and **Gaorong Han** initiated the work. **Zhaohui Ren**, **Gaorong Han**, **He Tian**, and **Tianqi Deng** designed the experiments and calculations and supervised the analysis of obtained results. **Yiran Sun**, **Chen Lin**, **Ruian Zhang**, and **Yi Fu** synthesized the samples, conducted the macroscopic morphology and structure characterization. **Yiran Sun** and **He Tian** completed the TEM studies, analyzed the TEM data and measured displacements of Ti ions. **Xingzhong Cao** and **Peng Zhang** performed PAS measurements. **Yiran Sun** and **Zihang Huang** carried out the photovoltaic measurement. **Jingjing Xue**, **Zhenbang Dai**, and **Andrew M. Rappe** contributed to the fitting model and its impact on the photovoltaic current. **Tianqi Deng** and **Xuanyu Jiang** performed the DFT calculations. **Junhui Wang** and **Kaifeng Wu** completed TA characterization and analysis. **Yiran Sun**, **Junhui Wang**, **Zhenbang Dai**, **Xingzhong Cao**, and **Zhaohui Ren** contributed to the analysis and understanding of the data and co‐wrote the manuscript. **Peng Zhang**, **Tianqi Deng**, **Kaifeng Wu**, **Andrew M. Rappe**, and **Gaorong Han** revised the manuscript. All authors participated in the analysis and discussion.

## Funding

This work was supported by the National Key R&D Program of China (2023YFA1406404), the National Natural Science Foundation of China (52272129, 22422307, 22273102, and 52502130), the Natural Science Foundation of Zhejiang Province, China (LR21E020004), the Shanxi‐Zheda Institute of Advanced Materials and Chemical Engineering (2021SX‐FR007), Fundamental Research Funds for the Central Universities (226‐2023‐00064), the Youth Innovation Promotion Association CAS (2021185), LiaoNing and Dalian Excellent Youth Science Fund (2025JH6/101100010, 2022RY30), Dalian Institute of Chemical Physics (DICP I202223, DICP I202410), U.S. Department of Energy, Office of Science, Basic Energy Sciences (DE‐SC0024942).

## Conflicts of Interest

The authors declare no conflicts of interest.

## Supporting information




**Supporting File**: advs76000‐sup‐0001‐SuppMat.docx.

## Data Availability

The data that supports the findings of this study are available in the supplementary material of this article.
