# Supplementary material for: Colossal Photovoltaic Current in Ferroelectric Oxide by Constructing Defect Band
Source: Adv Sci (Weinh). 2026 Jun 9:e76000. Online ahead of print. doi: 10.1002/advs.76000 (PMC13336417; doi:10.1002/advs.76000)
Supplement: Supplementary file 1 — Supporting File: advs76000‐sup‐0001‐SuppMat.docx. [file ADVS-9999-e76000-s001.docx]

Supporting Information

Colossal photovoltaic current in ferroelectric oxide by constructing defect band

*Yiran Sun, Zhenbang Dai, Xingzhong Cao, Peng Zhang, Chen Lin, Zihang Huang, Ruian Zhang, Yi Fu, Xuanyu Jiang, He Tian, Jingjing Xue, Kaifeng Wu, Junhui Wang*, Tianqi Deng*, Andrew M. Rappe, Gaorong Han*, Zhaohui Ren**

*Correspondence: Junhui Wang (wjh@dicp.ac.cn); Tianqi Deng (dengtq@zju.edu.cn); Gaorong Han (hgr@zju.edu.cn); Zhaohui Ren (renzh@zju.edu.cn).

**This file includes:**

**Methods**

**Supplementary Text**

**Figures S1–S28**

**Tables S1–S4**

**References** **53–81**

**Materials and Methods**

**Materials**

Lead nitrate (Pb(NO_3_)_2_, ≥99.0%), tetrabutyl titanate (TBOT, ≥98.0%), potassium hydroxide (KOH, ≥85.0%), acetone (≥99.5%) and ethanol (≥99.7%) were purchased from Sinopharm Chemical Reagent Co. Ltd. and used without further purification. single-crystal 0.7 wt.% Nb doped SrTiO_3_ (NSTO) substrates with dimensions 10 × 10 × 0.5 mm^3^ and (100) orientation were obtained from Shanghai Institute of Optics and Mechanics.

**Film preparation**

PTO films were synthesized by a hydrothermal method as follows: Pb(NO_3_)_2_ and TBOT were used as starting materials. KOH was used as a mineralizer. NSTO substrate was cleaned ultrasonically with acetone, ethanol and deionized water before using and then placed in a Teflon holder for drying. The Pb/Ti molar ratio was set at 1.25:1 and the concentration of KOH was 6M. KOH was first dissolved in deionized water, TBOT was then added in the aqueous solution, Pb(NO_3_)_2_ solution was finally dropwise added under vigorous stirring. After 2 h of continuous stirring at room temperature, the NSTO substrate was placed in the solution of the autoclave before hydrothermal treatment under 200°C for 12 h. NSTO substrate was maintained horizontally 15 mm above the bottom of the autoclave. The resultant products were washed with water and ethanol several times and subsequently dried at 60°C in air. For the annealed films, the following steps were taken: placing the samples in a porcelain boat for 2 h annealing in an oxygen atmosphere, and finally cooling to room temperature.

**Characterization**

The structures of the sample were characterized by scanning electron microscope (SEM, Hitachi SU-70 Analytical FESEM), X-ray Diffraction (XRD, Thermo ARL X’TRA powder diffractometer, Cu *Kα* radiation, *λ*=1.54056 Å), off-axis X-ray reciprocal space mapping (RSM, high-resolution thin film XRD Bruker D8 Discover), piezoelectric force microscope (PFM, Asylum Research, Cypher S and MFP-3D), conductive atomic force microscopy (c-AFM, Asylum Research, Cypher ES), X-Ray photoelectron spectroscopy (XPS, Thermo Scientific ESCALAB 250Xi) and electron paramagnetic resonance (EPR, Bruker A300 X-band EPR spectrometer). An integrated Focused Ion Beam (FIB, Helios nanolab 600) and SEM (Hatachi S4700) was used to extract thin-film lamellae from the PTO/NSTO sample, Pt and Au layers were deposited on film surface to protect from damage and increase the conductivity, respectively. Subsequent cross-sectional observations of the film were carried out using aberration-corrected transmission electron microscope (FEI Titan G2 80-200 Chemi STEM). The variation in composition across the thickness of PTO films were characterized by time-of-flight secondary-ion mass spectrometry (TOF-SIMS, IONTOF GmbH TOF.SIMS 5-100). The presence of lead vacancies was used by positron annihilation spectroscopy (PAS) based on slow positron facility in High Energy Accelerator Research Organization (KEK, Japan). The surface potential measurement was conducted in a Kelvin probe force microscopy (KPFM, Bruker) setup consisting of a modified Dimension Icon. The test of light absorption was conducted via a Cary 5000 UV-Vis-NIR Spectrophotometer.

**Transient absorption (TA) and transient reflection (TR)**

Femtosecond pump-probe transient absorption (TA) measurements were performed using a regenerative amplified Ti:sapphire laser system (Coherent; 800 nm, 70 fs, 6 mJ/pulse, and 1 kHz repetition rate) as the laser source and a femto-TA100 spectrometer (Time-Tech Spectra). Briefly, the 800 nm output pulse from the regenerative amplifier was split in two parts with a 50% beam splitter. The transmitted part was used to pump an OPA which generated a wavelength-tunable laser pulse from 250 nm to 2.5 μm as pump beam. The reflected 800 nm beam was split again into two parts. One part was attenuated with a neutral-density filter and focused into a 2-mm-thick sapphire or CaF_2_ window to generate a white light continuum (WLC) used for probe beam. The probe beam was focused with an Al parabolic reflector onto the sample. After the sample, the probe beam was collimated and then focused into a fiber coupled spectrometer with CMOS sensors and detected at a frequency of 1 kHz. The intensity of the pump pulse used in the experiment was controlled by a variable neutral-density filter wheel. The delay between the pump and probe pulses was controlled by a motorized delay stage. The pump pulses were chopped by a synchronized chopper at 500 Hz and the absorbance change was calculated with two adjacent probe pulses (pump-blocked and pump-unblocked). The transient reflection (TR) measurements were performed by using the same TA spectrometer with a slightly modified optical path in which the detection signal was changed from the transmitted probe light to the reflected probe light. And TR signal (ΔR/R) can also be determined by the ratio of the intensity of reflected probe light with and without pump excitation. All experiments were performed at room temperature.

**Photovoltaic measurement**

Photovoltaic properties of PTO films were collected using optical units from Thorlabs and source measure units (Keithley 2450). As shown in Figure S26a, the light from a monochromatic laser passes through a reflector and is vertically incident on the calibration detector (Optical power and energy meter consists of PM100USB and S130VC) or the sample surface. A diaphragm is used to control the on/off state of the light path. Top electrodes (diameter: ~1 mm, thickness: ~40 nm) were deposited on PTO surface by direct current (dc) sputtering (SD-3000). The schematic of the experimental setup for photovoltaic measurement was employed in Figure S26b. The films were illuminated with light from 266 nm (MPL-F-266), 375 nm (MDL-III-375), 405 nm (MDL-III-405), 532 nm (MGL-III-532) and 660 nm (MRL-FN-660) lasers.

**Calculations**

The density functional theory calculations were performed using the Vienna Ab initio Simulation Package (VASP) with projector-augmented-wave (PAW) method.^[53–55]^ The Perdew−Burke−Ernzerhof (PBE) functional ^[56]^ was employed for geometry optimization and electrostatic potential calculations, while the Heyd−Scuseria−Ernzerhof (HSE) hybrid functional ^[57,58]^ was employed for band structure calculations. The absorbance was computed in the independent particle approximation assuming a sample thickness of 200 nm. To account for the possibly disordered Pb vacancy, the absorption spectra of defective PTO with both ordered and random Pb vacancies were calculated and their average value was used for absorbance calculation. WannierTools ^[59]^ was used to perform electron band structure unfolding for defective PTO.

**Supplementary Text**

**Ⅰ: Physical background of the fitting formula for PV effect**

First, the junction-like effect is typically associated with the formation of p-n heterojunction or homojunction between different materials. This effect can be described by the Shockley diode equation, which accounts for the exponential dependence of current on voltage due to the barrier:^[31]^

$$\begin{aligned} J=J_{0}\cdot\left[ \exp\left( \frac{qV}{\eta kT} \right)-1 \right],\#\left( 1 \right) \end{aligned}$$

where *J*_0_ is the saturation current density, *k* is Boltzmann’s constant, *η* is the ideality factor, and *T* is the absolute temperature.

The anomalous PV (APV) effect, on the other hand, is related to the intrinsic property of non-centrosymmetric materials, such as ferroelectrics with a spontaneous polarization. This effect can be simply described by a linear relationship between current and voltage, reflecting the direct conversion of light into electrical current without the need for a junction in theory:^[32]^

$$\begin{aligned} J=J_{\mathrm{SC}}-\frac{V}{L}\left( \sigma_{d}+\sigma_{\mathrm{ph}} \right),\#\left( 2 \right) \end{aligned}$$

where *L* is the distance between electrodes, and σ_d_ and σ_ph_ are the dark and photoconductivity, respectively. Conventionally, APV can be explained by mechanisms such as shift current, injection current, or ballistic current. However, in practice, the observed PV behavior often extends beyond the ideal bulk PV effect mechanism. The composition gradient in PTO coupling with the spontaneous polarization may induce a gradient in polarization, which can also yield an approximately linear *J*-*V* response.^[25]^

The total PV current density *J* observed in our experiments could arise from a combination of both the junction-like effect and the APV effect. Therefore, the total fitting formula was written as:

$$\begin{aligned} J={AJ}_{0}\cdot\left[ \exp\left( \frac{qV}{\eta kT} \right)-1 \right]+B\cdot\left[ J_{\mathrm{SC}}-\frac{V}{L}\left( \sigma_{d}+\sigma_{\mathrm{ph}} \right) \right],\#\left( 3 \right) \end{aligned}$$

where *A* and *B* represent the proportion of junction and APV contribution, respectively. In the manuscript, four parameters a, b, c and d correspond to the ${AJ}_{0}$, $-\frac{\eta kT}{q}$, $BJ_{\mathrm{SC}}-{AJ}_{0}$ and $-\frac{B}{L}\left( \sigma_{d}+\sigma_{\mathrm{ph}} \right)$, respectively.

**Ⅱ: Extended discussion of optical absorption and bandgap characteristics in PTO films**

Here, we tried to grow the as-prepared and annealed PTO films on a La_0.7_Sr_0.3_MnO_3_ substrate under similar conditions and stripped the PTO films by a method reported in Ref. [39]. Then, we transfer the stripped samples to the transparent ITO glass for measuring light absorption using a Cary 5000 UV-Vis-NIR Spectrophotometer. Compared with the control film without annealing, the absorption of the annealed film increased significantly (Figure S21) in the range of 350–800 nm.

According to the UV-vis absorption spectra, we have fitted both the direct and indirect absorption edges using $\left( \alpha h\nu\right)^{2}$ and $\left( \alpha h\nu\right)^{1/2}$ versus $h\nu$ for the as-prepared and annealed films in Figure S21, respectively. For the as-prepared film, as shown in Figures S21b and S21c the obtained indirect gap is 2.66 eV, while the direct gap is 3.19 eV. This confirms the indirect band gap nature of PTO, which also agrees with the subsequent band structure calculations from first principles. The steep absorption edge around 400 nm, corresponds to the direct absorption contribution. The relatively flat absorption at wavelengths longer than 400 nm corresponds to the indirect absorption contribution.

For the annealed defective film, as shown in Figure S21d and S21e the obtained indirect gap is 2.46 eV, while the direct gap is 3.06 eV. It should be noted that the calculations only consider a pure Pb-deficient layer, whereas the tested films are composed of both the Pb-deficient and non-defective layers, which may explain why experimentally the annealed devices still exhibits indirect band gap. Although the defective PTO retains its characteristic indirect bandgap semiconductor behavior, the defect states induce additional direct absorption, significantly enhancing absorption efficiency at wavelengths longer than 400 nm. Only considering direct transitions, the theoretical absorption spectra already exhibit a seemingly flat absorption, as shown in Figure 3g. As illustrated in Figure 3f, the defect states induced possible direct transitions that span over a wide range of energy. This diversity of defect-induced transitions leads to the seemingly flat absorption. Therefore, this flat absorption edge could be explained by the additional direct transitions induced by the defect states.

In our calculation results, the band gaps of pristine PTO and defective PTO with 1/8 Pb vacancy were ≈2.925 eV and ≈1.146 eV, respectively. One the one hand, DFT calculations will underestimate the band gap. On the other hand, the calculations only consider a pure Pb-deficient layer, whereas the tested films are composed of both the Pb-deficient and non-defective layer, which may explain the difference between the theoretical and experimental absorption spectra. Additionally, we need to emphasize that the band gap obtained from the absorption spectrum is not the intrinsic band gap of the film in our experiment. As Figure 3f shows, the defect states formed by Pb vacancies break symmetry, enabling previously forbidden transitions at point Z/Γ and enhancing absorption with lower excitation energy. Such defect-related transitions dominate the absorption edge, leading to an apparent reduction in the experimental optical bandgap compared to the theoretical bulk value (≈2.9 eV). Therefore, we would like to argue that the measured optical band gap reduction is consistent with defect-induced transitions rather than a true narrowing of the intrinsic band gap of film.

**Ⅲ: Photocurrent calculation for perfect PTO with a built-in electric field**

Here, we are estimating how much photocurrent from the traditional photovoltaic (PV) effect will contribute to the total photocurrent observed in the PTO film, and demonstrating what factors will influence the magnitude of the photocurrent.

The reason for the existence of a traditional PV photocurrent is the gradual change of the polarization throughout the PTO film, which will generate a built-in electric field. On the other hand, since the film is rather thick (on the order few hundred nanometers), we will assume that the optical and transport properties of this graded film is the same as the perfect PTO, and therefore, we can calculate these optical and transport properties from first principles by using a single unit cell. Furthermore, the built-in electric field will be inferred from the experimentally resolved Ti-displacements.

Due to the existence of the built-in electric field, we propose to calculate the photocurrent from the drift-current formula:

$$\begin{aligned} J_{\mathrm{SC}}\left( \omega\right)=e\left( n_{e}\mu_{e}+n_{h}\mu_{h} \right)E,\#(4) \end{aligned}$$

where $n_{e}(n_{h})$ are the electron (hole) concentration in the unit of cm^−3^, $\mu_{e}(\mu_{h})$ are the electron (hole) mobility in the unit of cm^2^·V^−1^·s^−1^, $E$ is the built-in electric field in the unit of V·cm^−1^, and $e$ is the elementary charge in the unit of Coulomb. As a result, the unit of $J_{\mathrm{SC}}$ is A/cm^2^. If we make the approximation that the carriers are generated purely from the photo-excitation, then the carrier concentrations can be computed by assuming that every absorbed photon is converted to an electron-hole pair. Mathematically, they are

$$\begin{aligned} n_{e}=W_{tot}\left( \omega\right)\cdot\tau_{e}, n_{h}=W_{tot}\left( \omega\right)\cdot\tau_{h}.\#(5) \end{aligned}$$

Here, $W_{tot}\left( \omega\right)$ is the carrier generation rate due to the absorption of a photon with frequency$\omega$, and $\tau_{e}(\tau_{h})$ are the lifetimes of the carriers. Since the carrier generation is due to the optical absorption, we can write $W_{tot}\left( \omega\right)$ as

$$\begin{aligned} W_{tot}\left( \omega\right)=\frac{1}{8\pi\hbar}E_{0}^{2}{\varepsilon_{2}\left( \omega\right)\varepsilon}_{0},\#(6) \end{aligned}$$

where $E_{0}$ is the electric field of the light, and should not be confused with the built-in electric field $E$ in Eq. (4).^[60]^ $\varepsilon_{2}\left( \omega\right)$ is the imaginary part of the dielectric function, which is directly related to optical absorption, and $\varepsilon_{0}$ is the vacuum permittivity. Note that units of the electric field, $\varepsilon_{2}$, $\varepsilon_{0}$, and $\tau_{e}(\tau_{h})$ are V/cm, dimensionless, C^2^·kg^−1^·m^−3^·s^2^, and s, so the unit of $n_{e}(n_{h})$ is cm^−3^, as expected.

The electric field of light $E_{0}$ is ultimately an experimental parameter, which is determined by the light intensity according to the following relation:

$$\begin{aligned} \varepsilon_{0}E_{0}^{2}=\frac{2I}{c},\#(7) \end{aligned}$$

where $I$ is the light intensity and $c$ is the speed of light. Then, using Eqs. (5), (6) and (7), we can rewrite Eq. (4) as

$$\begin{aligned} j_{sc}\left( \omega\right)=e\left( n_{e}\mu_{e}+n_{h}\mu_{h} \right)E=e\frac{I}{4\pi\hbar c}\varepsilon_{2}\left( \omega\right)\left( \mu_{e}\tau_{e}+\mu_{h}\tau_{h} \right)E.\#(8) \end{aligned}$$

From this expression, it is already clear that the photocurrent will be proportional to $\varepsilon_{2}$, i.e., how large the optical absorption is. Experimentally, if one can engineer the system to enhance the optical absorption, create a stronger built-in electric field, or prolong the carrier lifetime, then it is expected to generate larger photocurrent from this material.

In order to evaluate Eq. (8) quantitatively, we use density functional theory (DFT) to calculate $\varepsilon_{2}\left( \omega\right)$. A scissors shift is used to match the experimental bandgap. The following expression is adopted:

$$\begin{aligned} \varepsilon_{2}\left( \omega\right)=\frac{16\pi e^{2}}{\omega^{2}}\sum_{vc} \left| \left\langle v | v | c \right\rangle\right|\delta\left( \omega-E_{c}+E_{v} \right),\#\left( 9 \right) \end{aligned}$$

where we set $\hbar=1,$ $\left\langle v | v | c \right\rangle$ is the velocity matrix between conduction band *c* and valence band *v*, and $E_{c}$ and $E_{v}$ are the electronic energies of the state *c* and *v*, respectively.^[61]^ We sample these quantities in a 24 × 24 × 24 ***k***-grid, and the calculations are performed in Quantum Espresso.^[62,63]^

We compute the phonon-limited carrier lifetime and solve the Boltzmann transport equation (BTE) using the EPW package.^[64]^ An 8 × 8 × 8 Brillouin-zone grid is used to sample the electronic states, phonon modes, and electron-phonon coupling matrix elements, while these quantities are subsequently interpolated to a 60 × 60 × 60 to compute the carrier lifetimes and solve the BTE. We average the lifetimes of all electrons and holes within a 25 meV energy window above the conduction band minimum and valence band maximum, respectively, which gives

$$\begin{aligned} \tau_{e}=9.3 fs, \tau_{h}=56.4 \mathrm{fs},\#\left( 10 \right) \end{aligned}$$

and the electron and hole mobilities at room temperature are

$$\begin{aligned} \mu_{e}=19.9 cm^{2}V^{-1}s^{-1}, \mu_{e}=95.6 cm^{2}V^{-1}s^{-1}.\#\left( 11 \right) \end{aligned}$$

The built-in electric field is estimated from experimentally measured displacements of Ti at the interface and surface. Specifically, considering the sharp change of the Pb concentration near the annealed PTO/NSTO interface (Figure 2c), we assume that most of the polarization change happens near the interface within ≈ 10 nm. With the given displacement of Ti, we can compute the corresponding electric polarization using the Berry phase approach.^[65]^ Assuming that change of the polarization near the interface changes as a hyperbolic tangent, then we will have the polarization profile $P\left( x \right)$, as shown in Figure S27. With $P\left( x \right)$, we can solve the Poisson’s equation^[66]^

$$\begin{aligned} \frac{d^{2}V\left( x \right)}{dx^{2}}=-\frac{1}{\varepsilon}\frac{dP\left( x \right)}{dx},\#\left( 12 \right) \end{aligned}$$

from which we obtain the change of potential $\Delta V$ across the film thickness *L*:

$$\begin{aligned} \Delta V=-\frac{1}{\varepsilon}\int_{0}^{L} \left[ P\left( x \right)-P\left( 0 \right) \right]dx.\#\left( 13 \right) \end{aligned}$$

Then, the average electric field across the film approximated to be

$$\begin{aligned} E\approx\frac{\Delta V}{L}=1.1\times{10}^{8} V/m.\#\left( 14 \right) \end{aligned}$$

With the carrier lifetimes, the mobilities, and the built-in electric field, we proceed to compute the photocurrent from the traditional PV in a perfect PTO, as shown in the Figure S28. We found that for light intensity *I*_light_ = 500 mW/cm^2^, the computed photocurrent at 375 nm (3.31 eV) is only 2.2 mA/cm^2^, which is smaller than the experimental value by a factor of 15 (Figure 3a). In fact, this value is similar to the photocurrent observed in the pure PTO without annealing.^[25]^ Therefore, for a pure PTO, one cannot achieve as high photocurrent as what we found in experiments, and it supports the hypothesis that the surface layer generated by annealing will enhance the optical absorption, strengthen the built-in electric field, and prolong the carrier lifetimes, which will in turn enhance the photocurrent.

**Figures and tables**


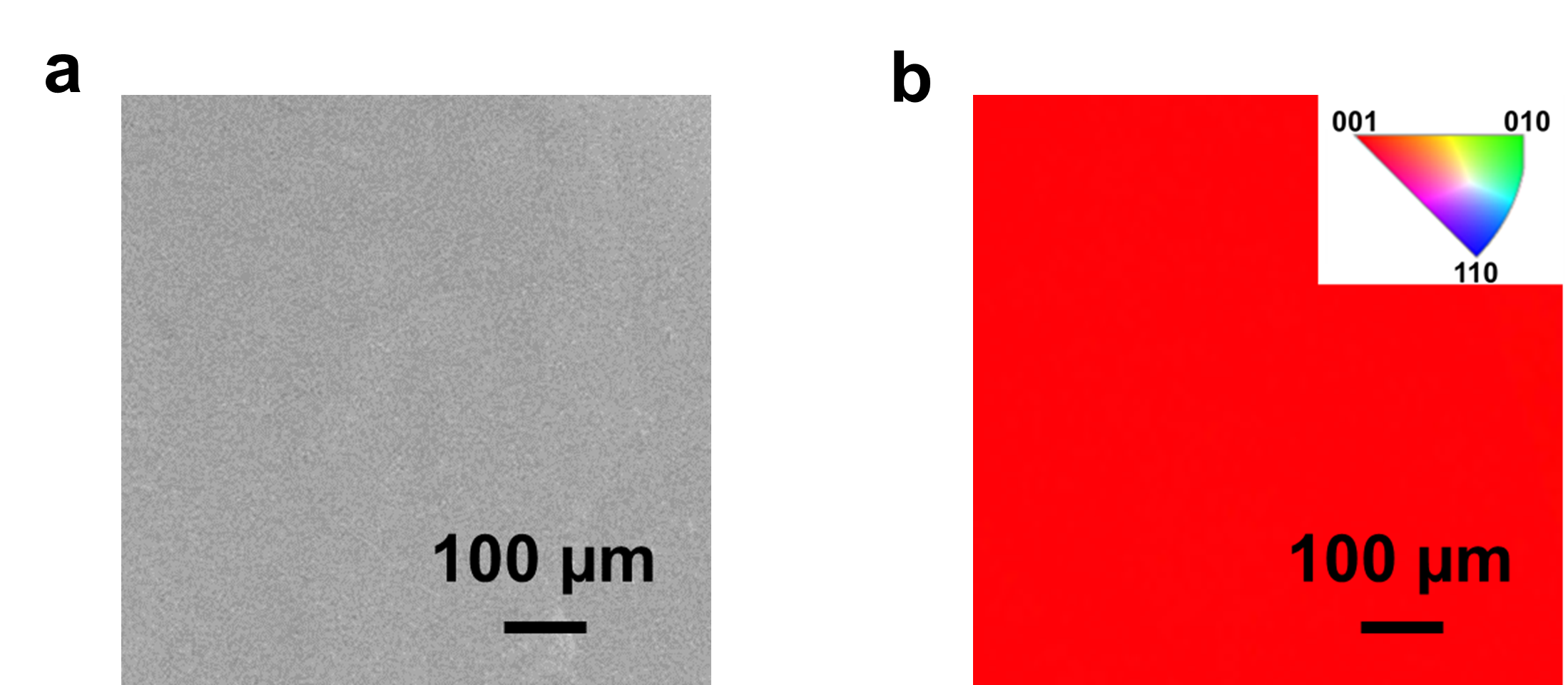


**Figure S1. a,** EBSD band contrast image of as-prepared PTO film. **b,** The corresponding EBSD IPF Z direction color map. The legend for interpreting the IPF map is shown in the inset.


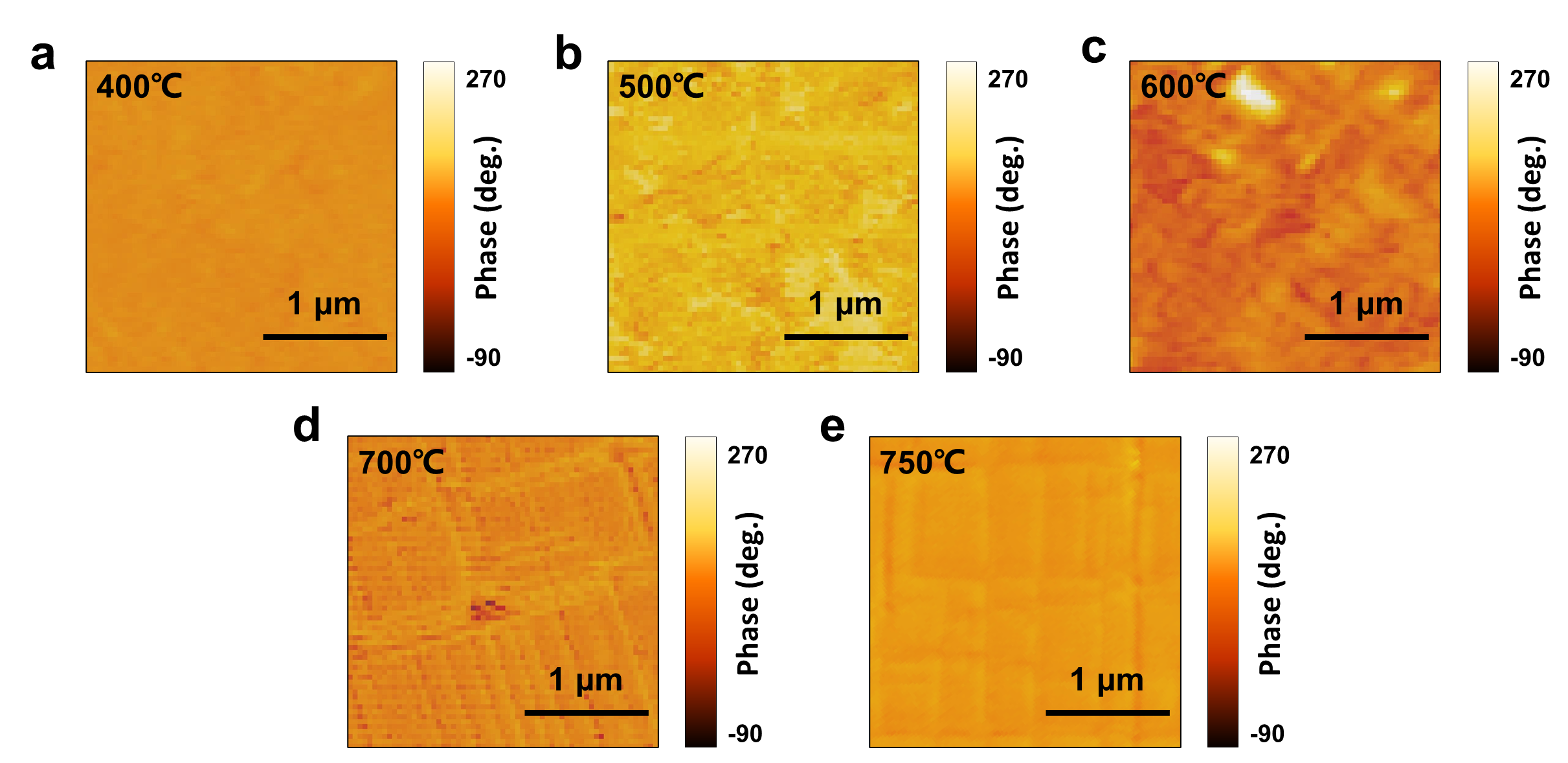


**Figure S2. a–e,** Out-of-plane PFM phase images of PTO films annealed at (a) 400°C, (b) 500°C, (c) 600°C, (d) 700°C and (e) 750°C.


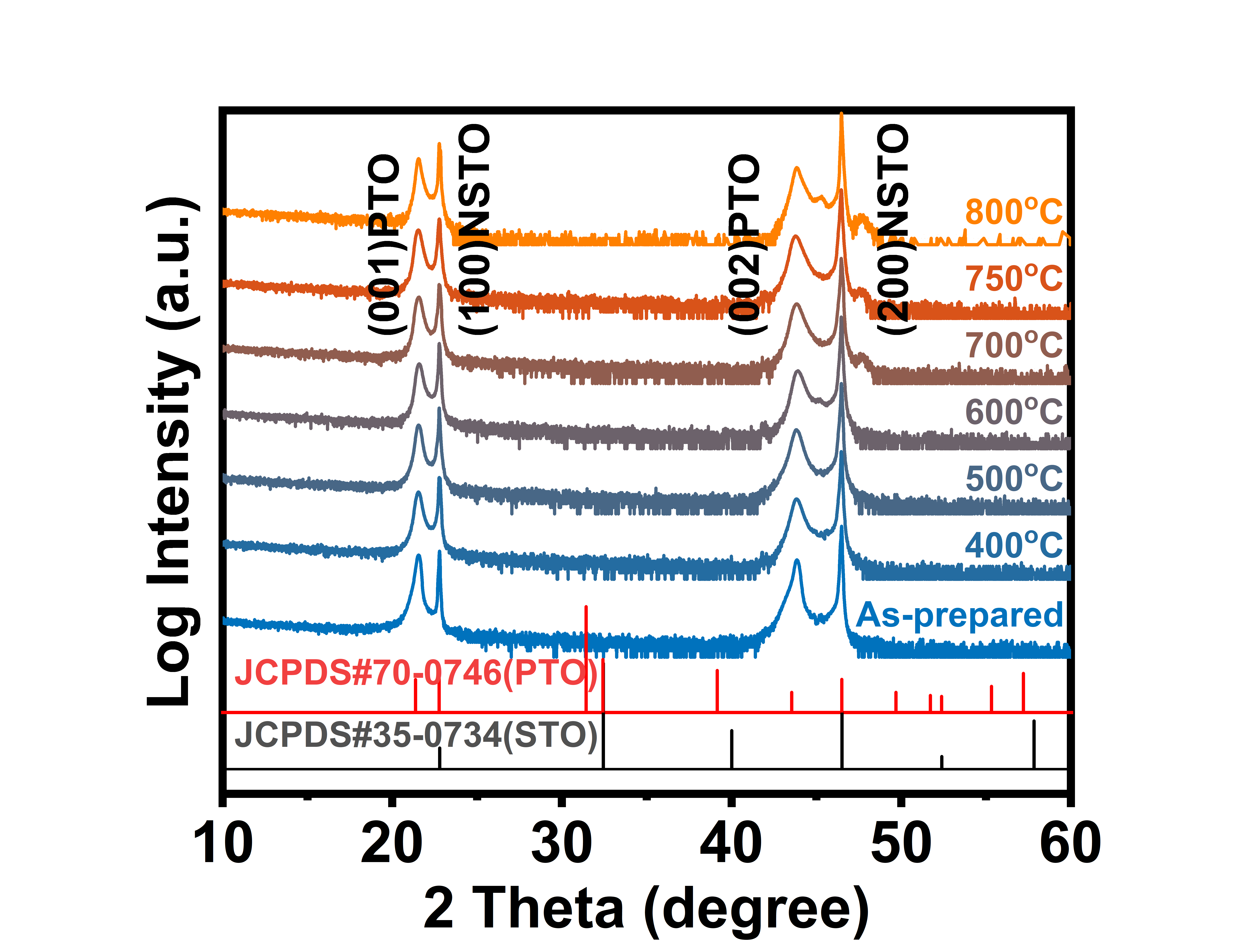


**Figure S3.** X-ray diffraction (XRD) patterns in log scale of PTO films annealed at different temperatures.


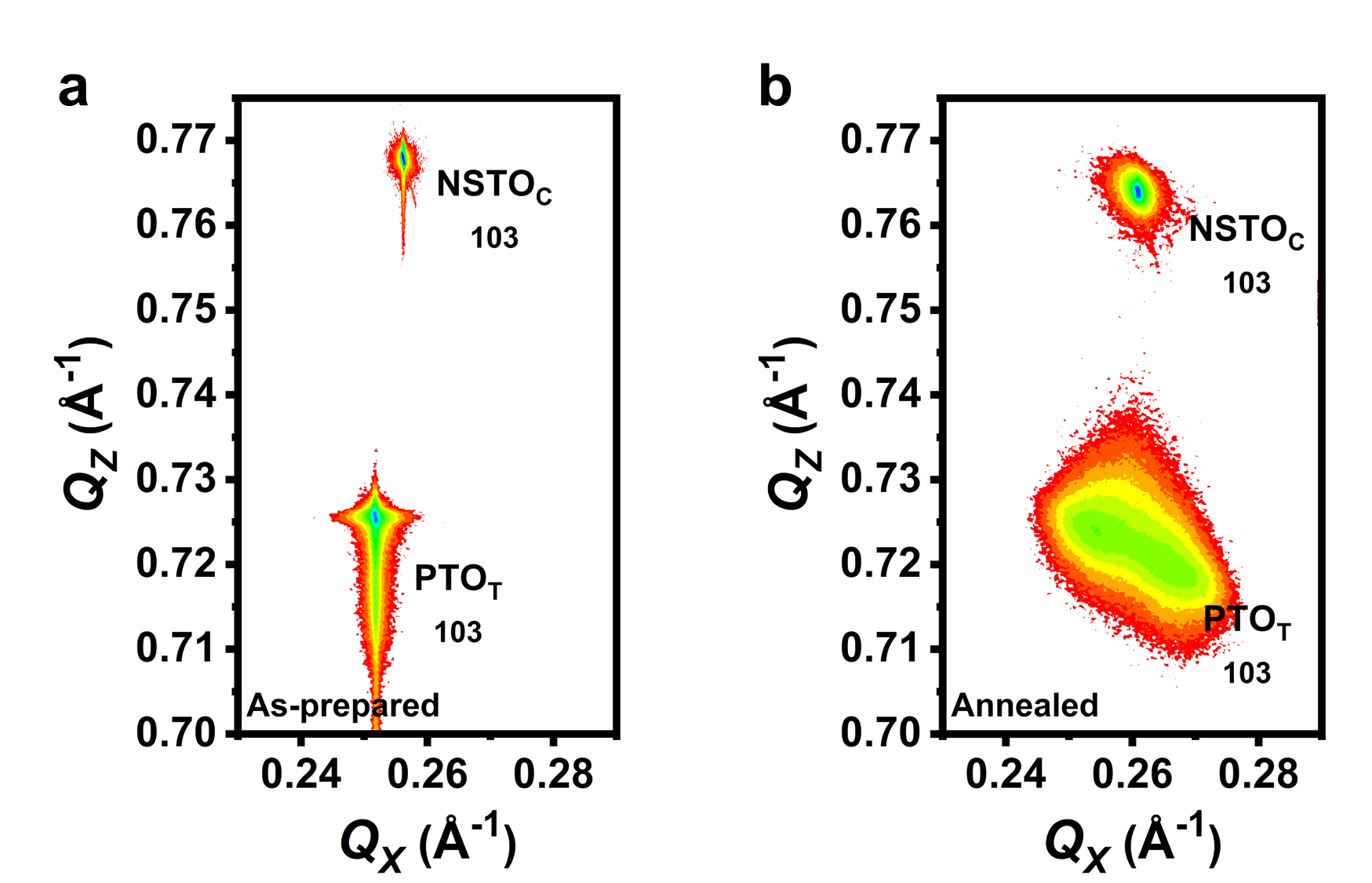


**Figure S4. a,b,** RSM of (a) as-prepared and (b) annealed (750℃) PTO films.


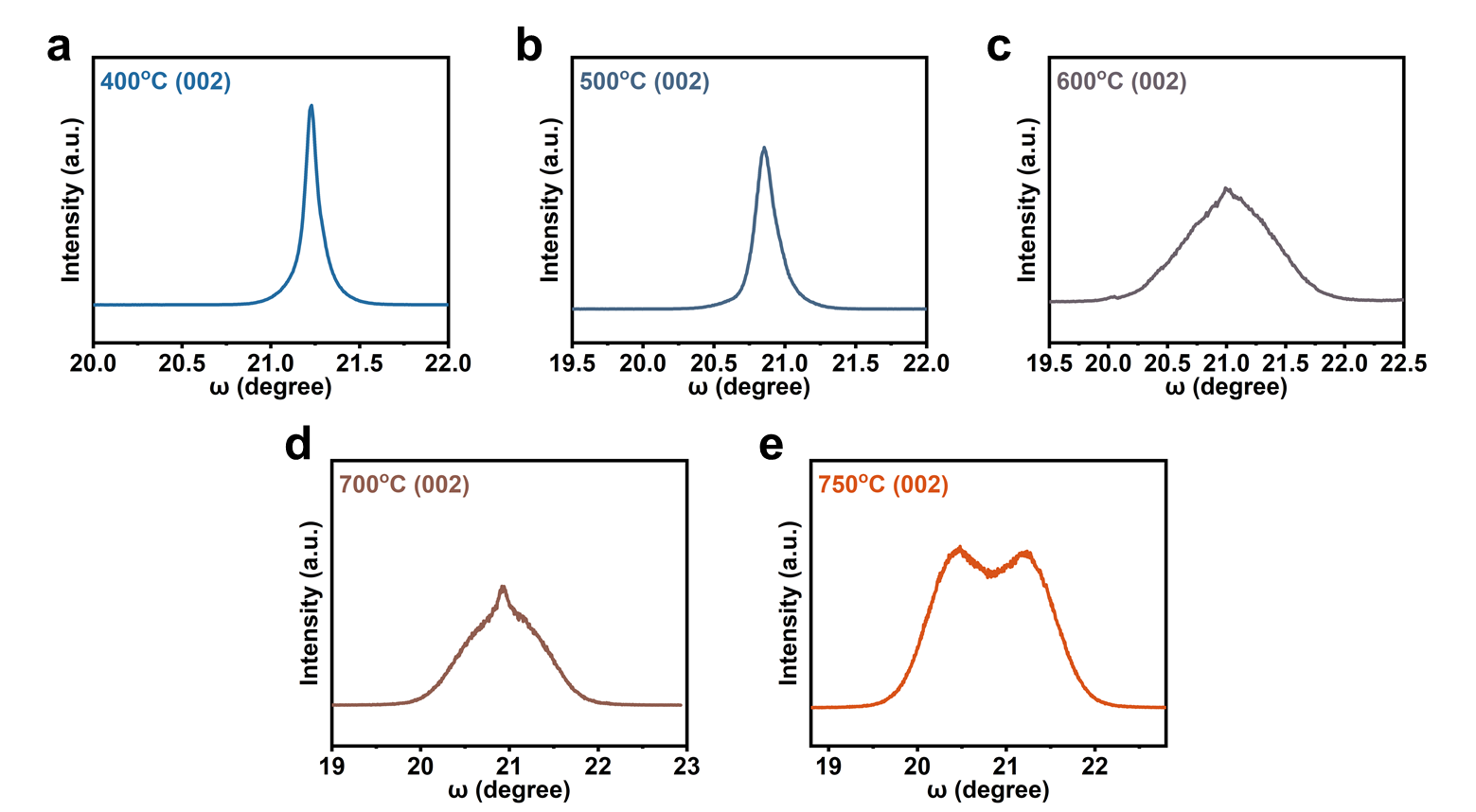


**Figure S5. a–e,** X-ray rocking curves of PTO films annealed at (a) 400°C, (b) 500°C, (c) 600°C, (d) 700°C and (e) 750°C.


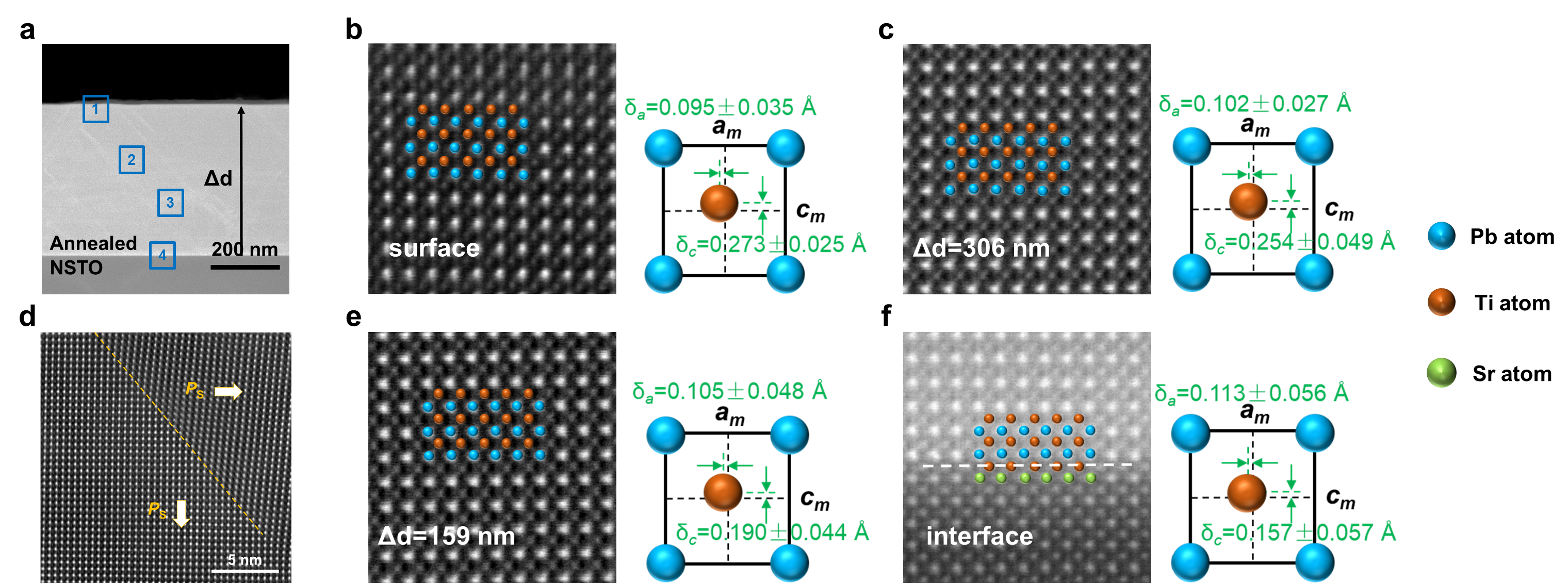


**Figure S6. a,** Cross-sectional HAADF-STEM image of annealed (750℃) PTO film, where ∆*d* respectively represents for the distances from the interface. **b,c,e,f,** Atomic-scale cross-sectional HAADF-STEM images of regions with different ∆d in annealed PTO film: (b) ∆*d* = 445 ‍nm, (c) ∆*d* = 306 nm, (e) ∆*d* = 159 nm, (f) ∆*d* = 0 nm, corresponding to blue boxes 1–4 in (a). Insets are the schematic illustrations of the average-displacement measurement of Ti^4+^ relative to the center of the four nearest Pb^2+^ in each of the corresponding regions, where the δ*_a_* and δ*_c_* respectively denotes the off-center displacements of Ti^4+^ along *a*-axis and *c*-axis. **d,** Atomic-scale cross-sectional HAADF-STEM images of annealed PTO film containing 90° and 180° domains.


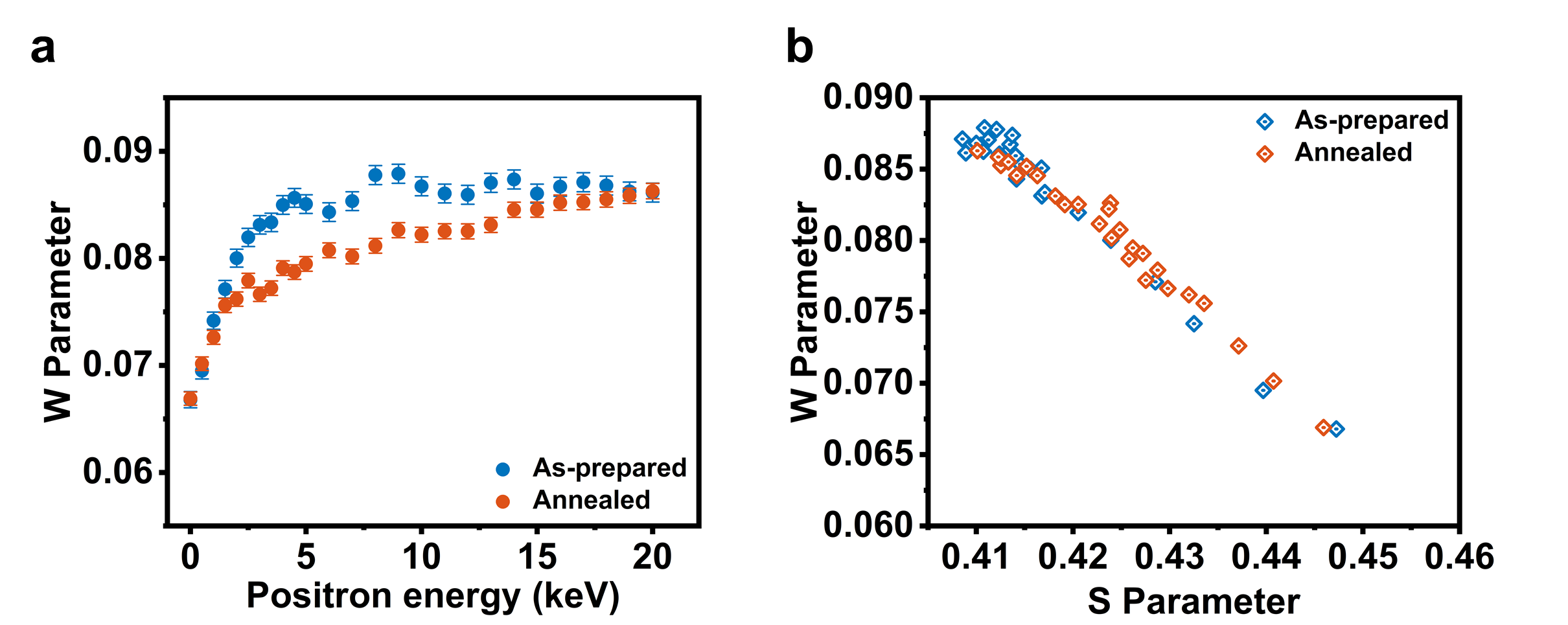


**Figure S7. a,** VE-DBS W-parameter versus positron implantation energy of as-prepared and annealed (750℃) PTO films. **b,** S-W curve of as-prepared and annealed films using the 499.5–522.5 keV spectra from the VE-DBS measurements.


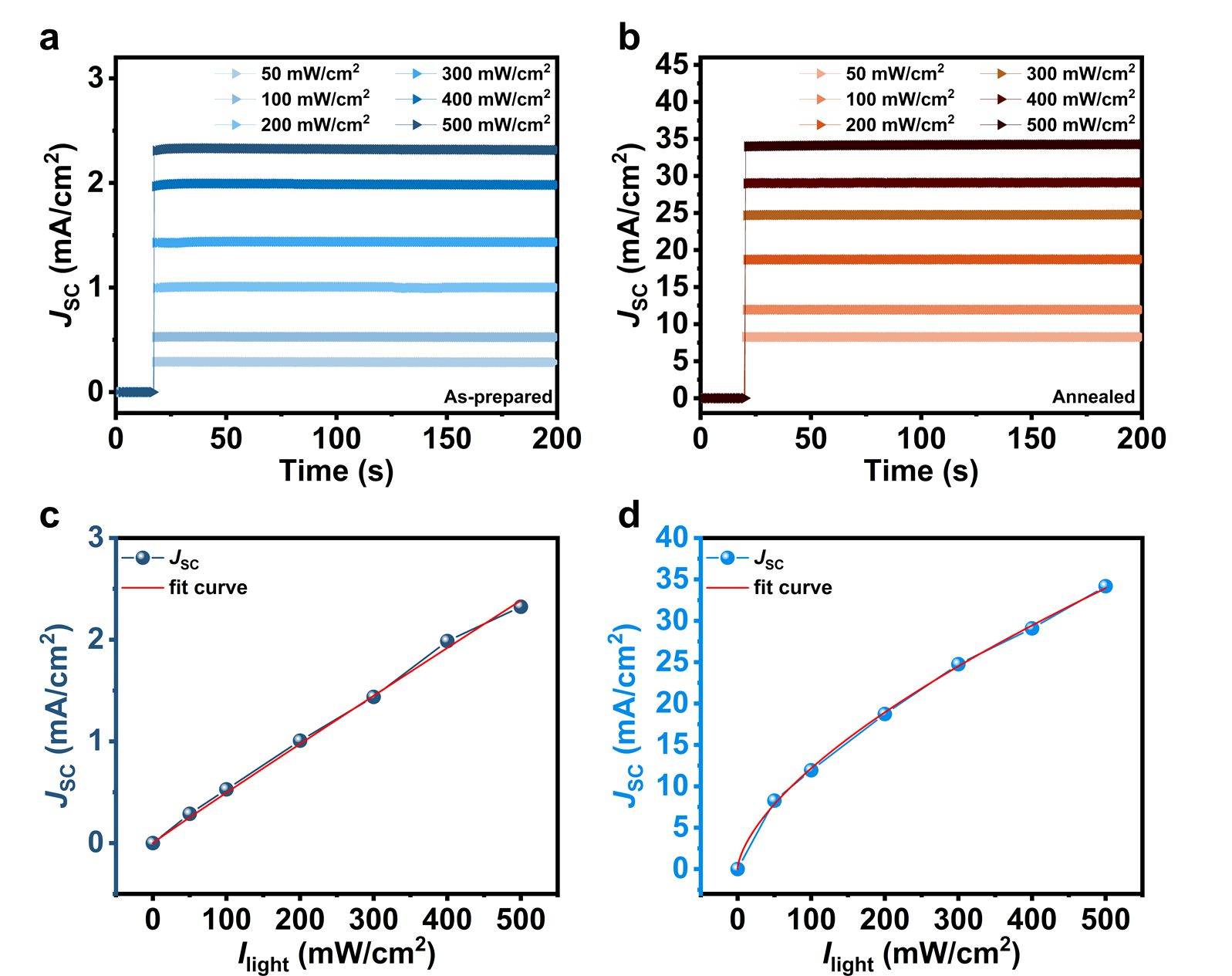


**Figure S8. a,b,** Steady state short-circuit current density of (a) as-prepared and (b) annealed PTO films at zero bias under the illumination of different *I*_light_ from a 375 nm laser. **c,d,** Fitting results of *J*_SC_-*I*_light_ curve for (c) as-prepared and (d) annealed PTO films.


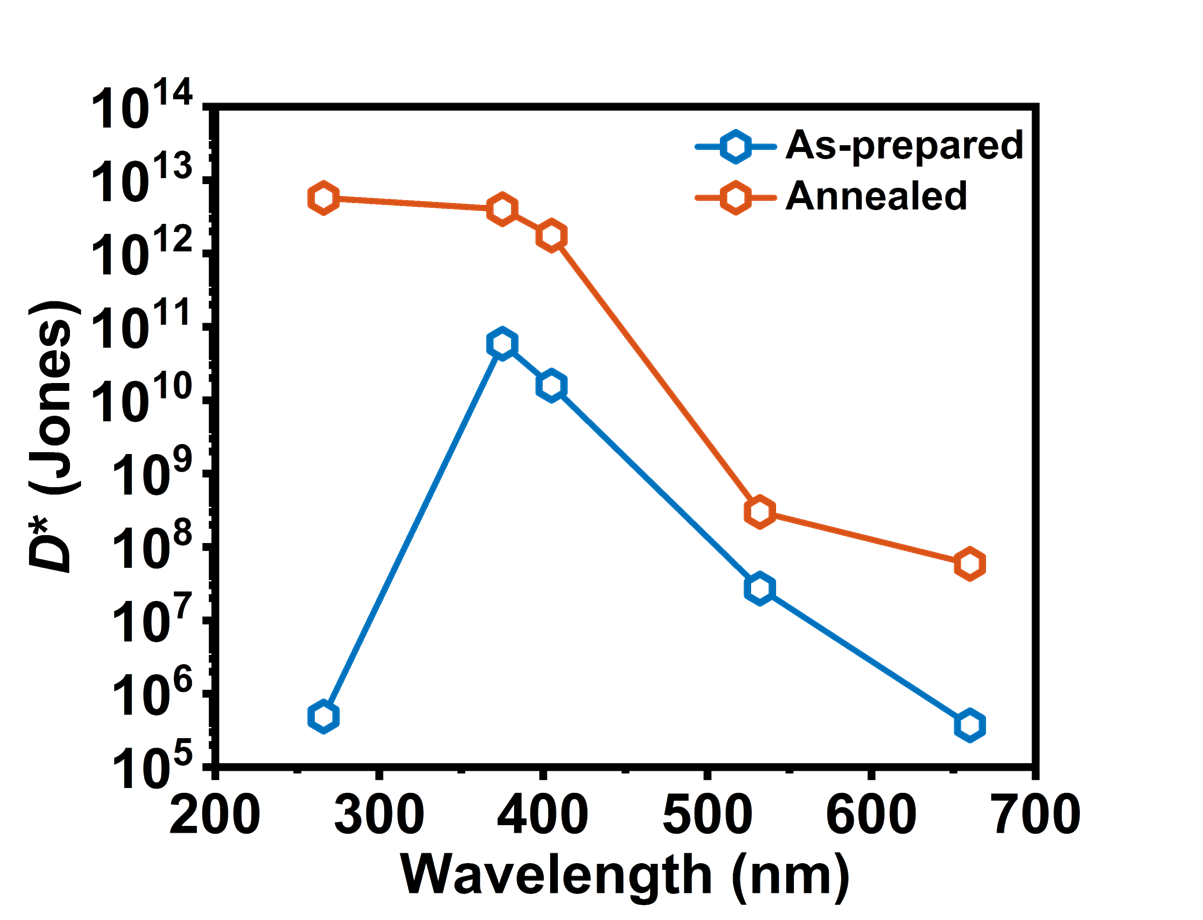


**Figure S9.** Detectivity (*D**)-wavelength curve of as-prepared and annealed (750℃) PTO films.


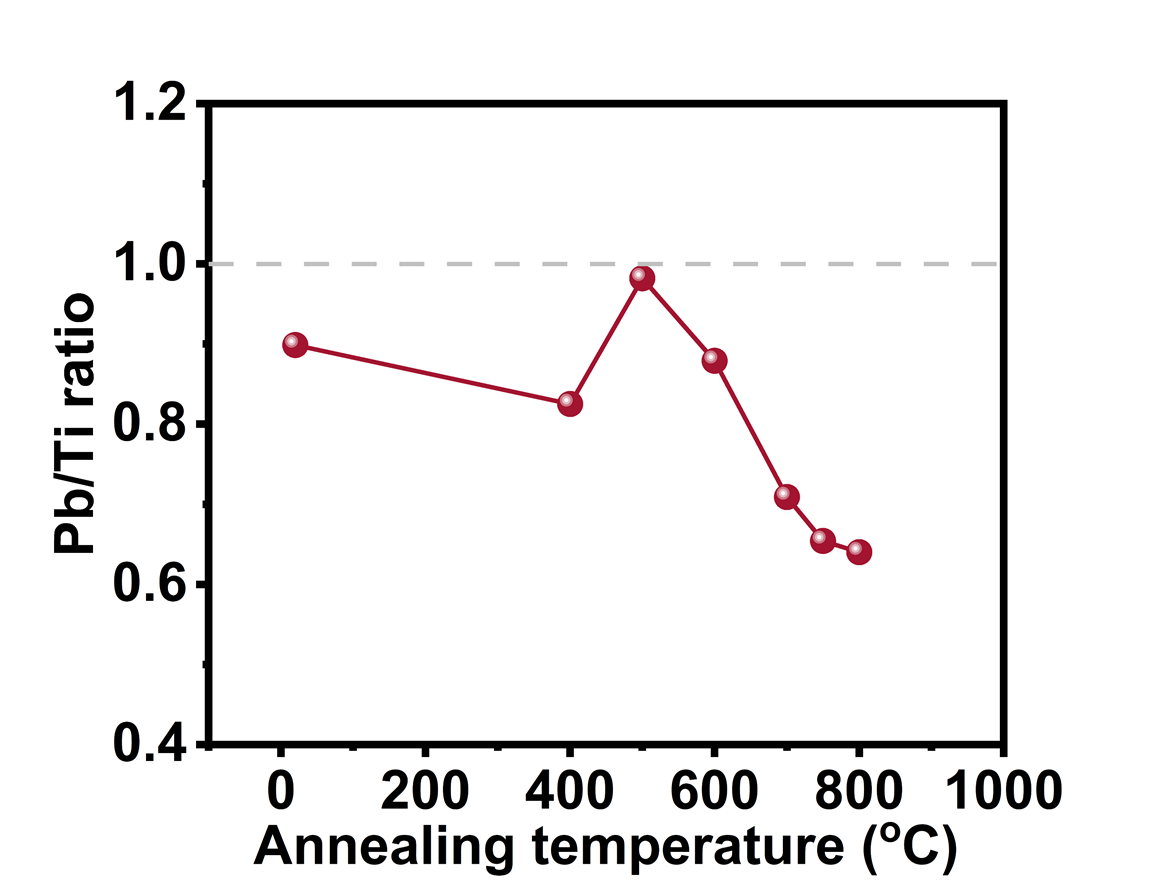


**Figure S10.** Pb/Ti ratio of PTO films annealed at different temperatures calculated from XPS results.


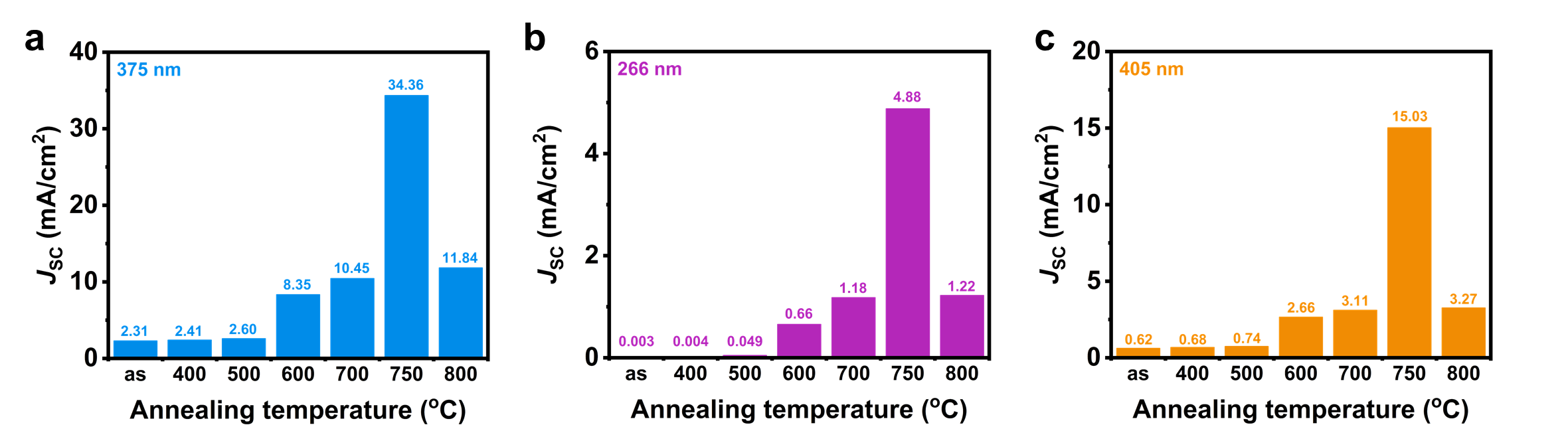


**Figure S11. a–c,** Steady-state short-circuit current density of PTO films annealed at different temperatures under the fixed illumination from (a) 375 nm (*I*_light_ = 500 mW/cm^2^), (b) 266 nm (*I*_light_ = 50 mW/cm^2^) and (c) 405 nm lasers (*I*_light_ = 500 mW/cm^2^).


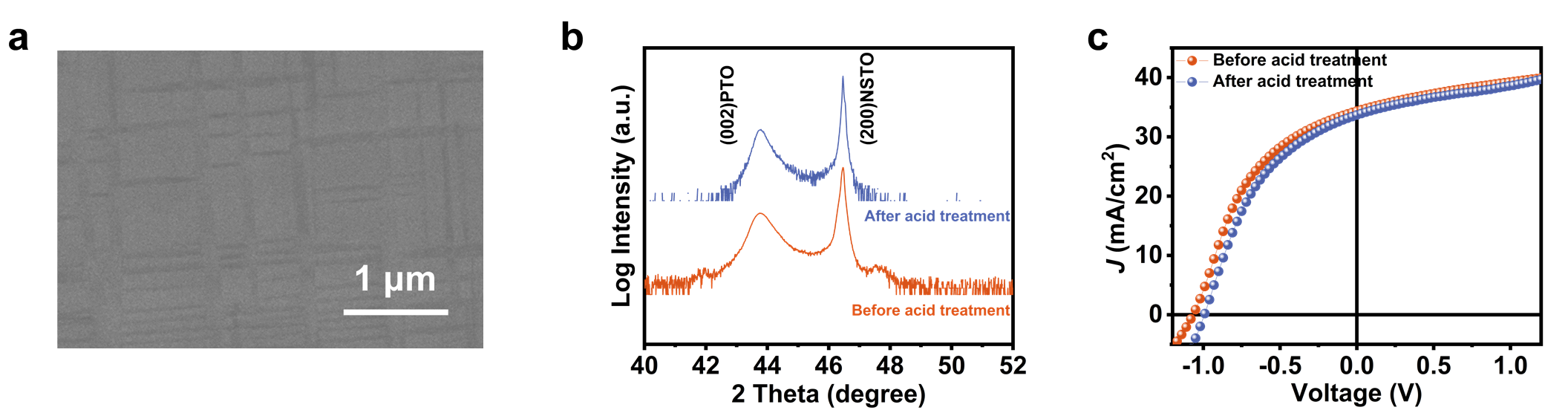


**Figure S12. a,** SEM image of annealed (750℃) PTO film with acid treatment. **b,** XRD patterns of annealed PTO film before and after acid treatment. **c,** *J-V* curves of annealed PTO film before and after acid treatment under the illumination of 500 mW/cm^2^ *I*_light_ from a 375 nm laser.


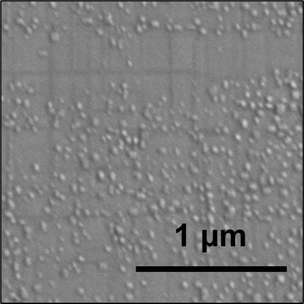


**Figure S13.** SEM image of PTO film annealed at 800℃.


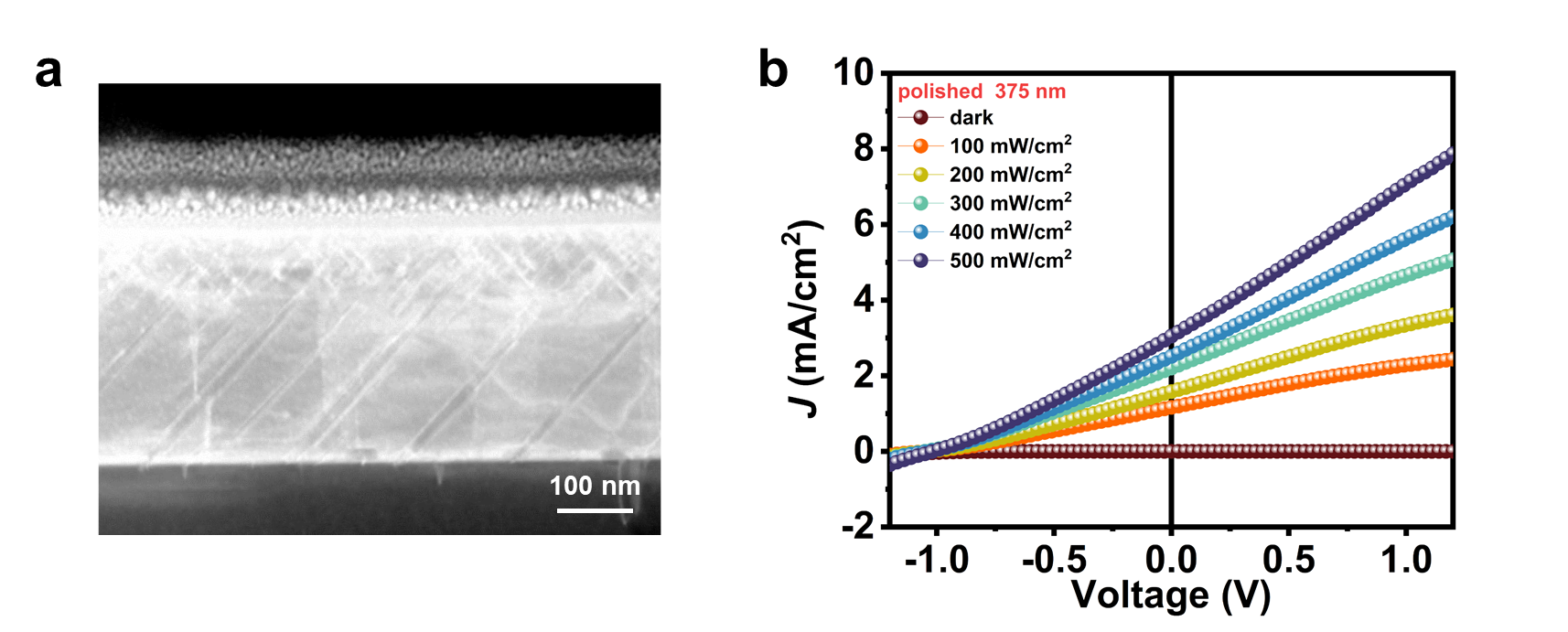


**Figure S14. a,** Cross-sectional HAADF-STEM image of annealed (750℃) PTO film after polishing. **b,** *J-V* curve of annealed PTO film after polishing under the illumination of different *I*_light_ from a 375 nm laser and under dark condition.


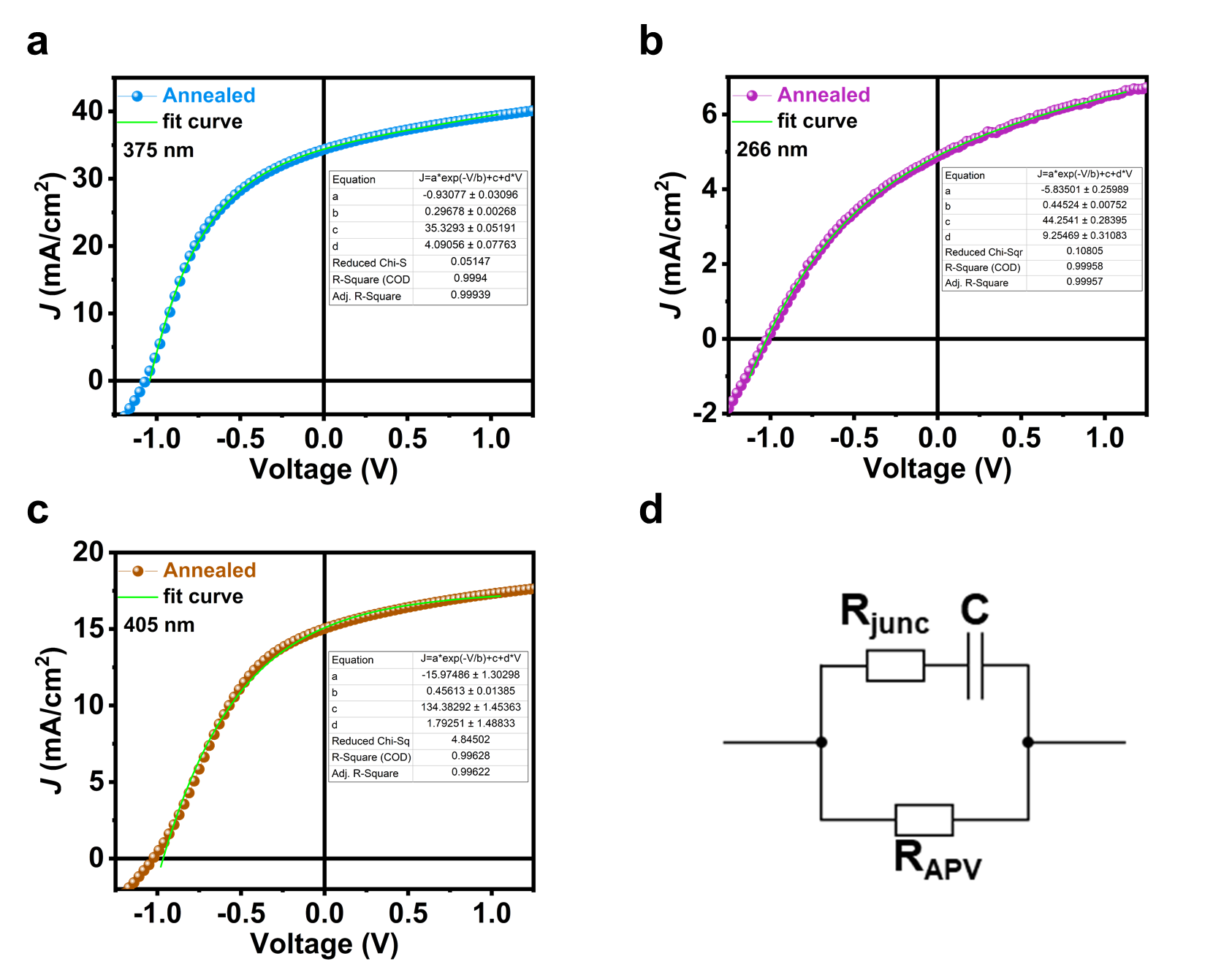


**Figure S15. a–c,** Fitting results of *J-V* curves for annealed (750℃) PTO film under the illumination of (a) 375 nm, (b) 266 nm and (c) 405 nm lasers. **d,** The schematic circuit diagram of annealed PTO film. R_junc_, R_APV_ and C represent the resistance of the junction, the resistance of the APV effect and the capacitance, respectively.


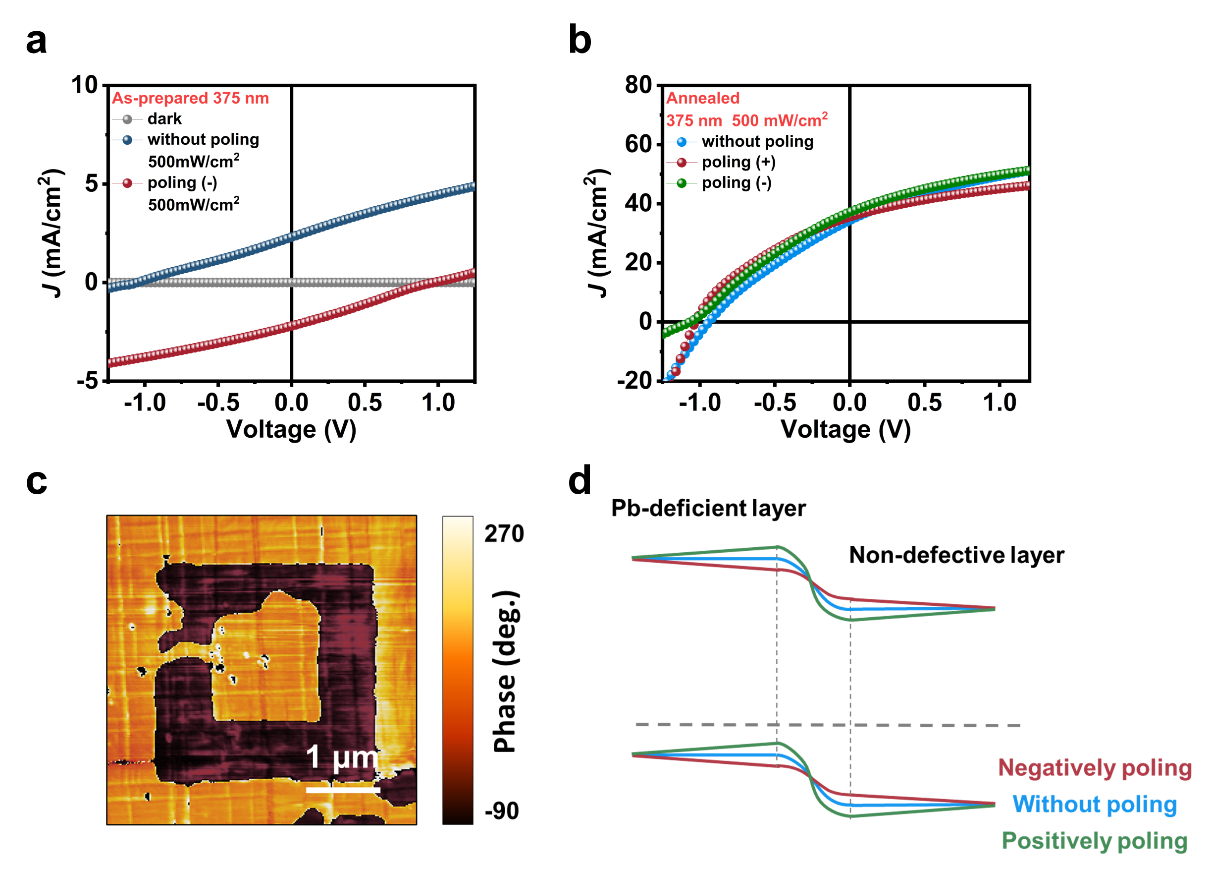


**Figure S16. a,** *J*-*V* curves of as-prepared PTO film without poling and with a negative poling under the illumination of 500 mW/cm^2^ *I*_light_ from a 375 nm laser as well as under dark condition. **b,** *J*-*V* curves of annealed PTO film without poling, with positively and negatively poling under the illumination of 500 mW/cm^2^ *I*_light_ from a 375 nm laser as well as under dark condition. **c,** Out-of-plane PFM phase images recorded after the box-in-box writing process. In the writing process, a tip bias of -10 V was firstly applied on a 3×3 ‍μm^2^ squared area and followed by a tip bias of +10 V on the central 1.5×1.5 μm^2^ area. **d,** Schematic of the energy band diagram of annealed film without poling, with positively and negatively poling.


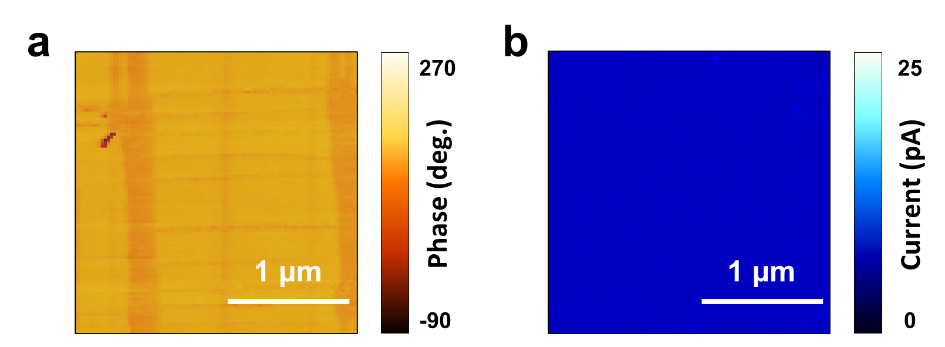


**Figure S17.** **a,b,** (a) Out-of-plane PFM phase image and (b) c-AFM map of the annealed film under dark condition.


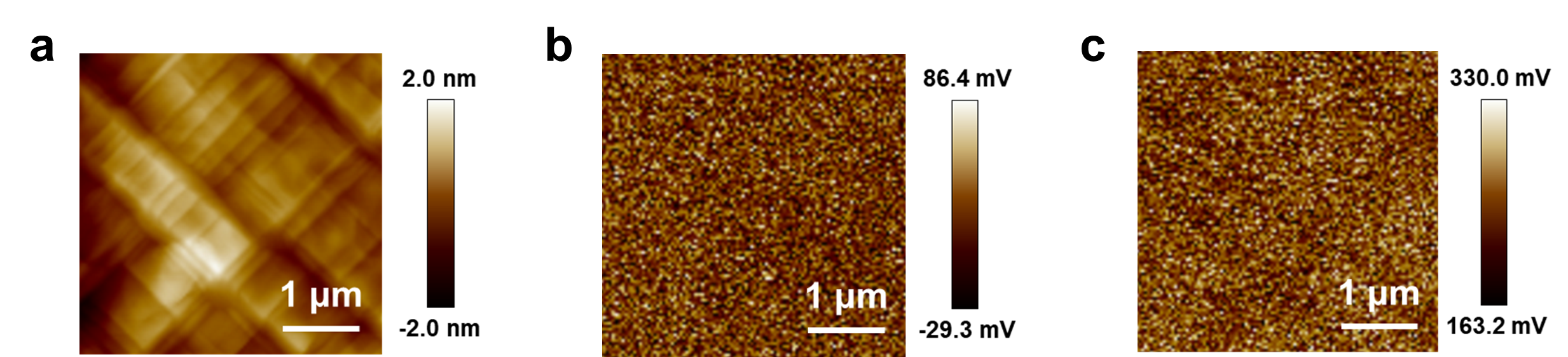


**Figure S18.** **a**, Topography image of annealed film. **b**, Surface potential maps of annealed film under dark condition. **c**, Differential surface potential map of annealed film obtained by subtracting the dark-state potential from the illuminated-state potential.


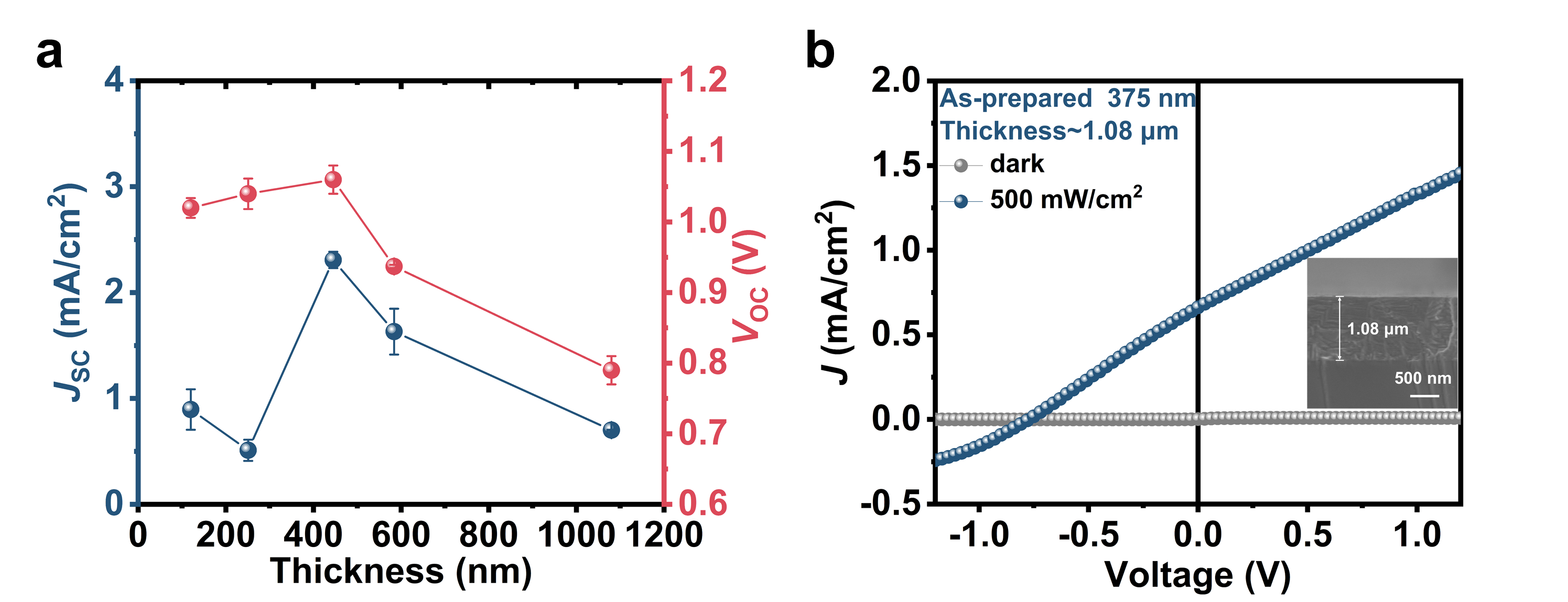


**Figure S19.** **a**, *J*_SC_ and *V*_OC_ under the illumination of 500 mW/cm^2^ *I*_light_ from a 375 nm laser as a function of PTO film thickness. **b**, *J*-*V* curve of as-prepared PTO film with a thickness of ≈1.08 μm under the illumination of 500 mW/cm^2^ *I*_light_ from a 375 nm laser as well as under dark condition. The inset is cross-sectional SEM image of PTO film with a thickness of ≈1.08 μm.


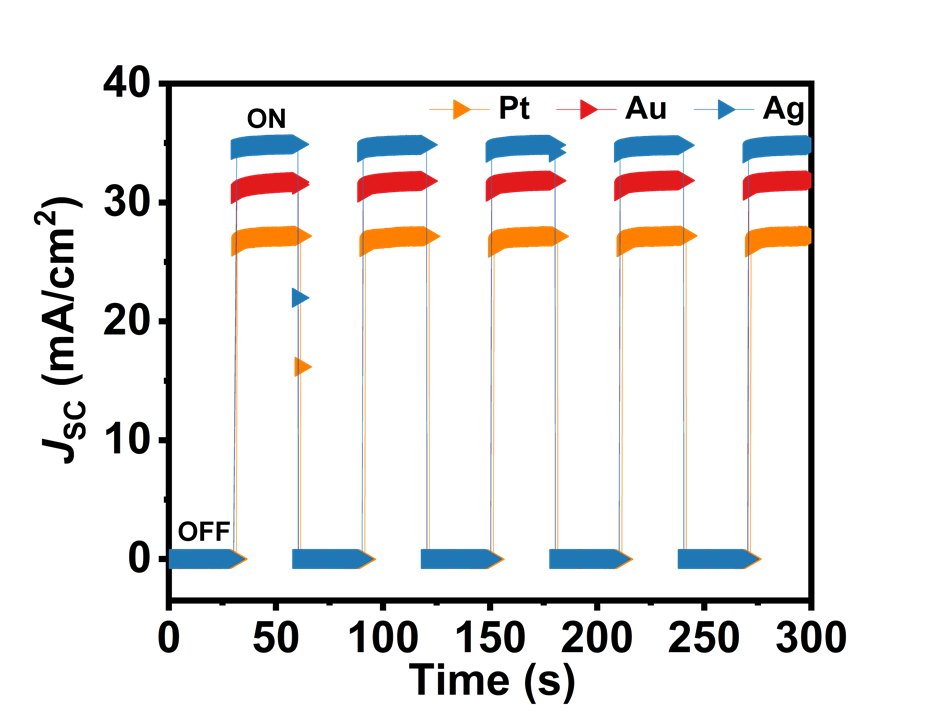


**Figure S20.** Short-circuit current density of annealed (750℃) PTO film response to the switching of a light on and off at zero bias with different top electrode under the illumination of 500 mW/cm^2^ *I*_light_.


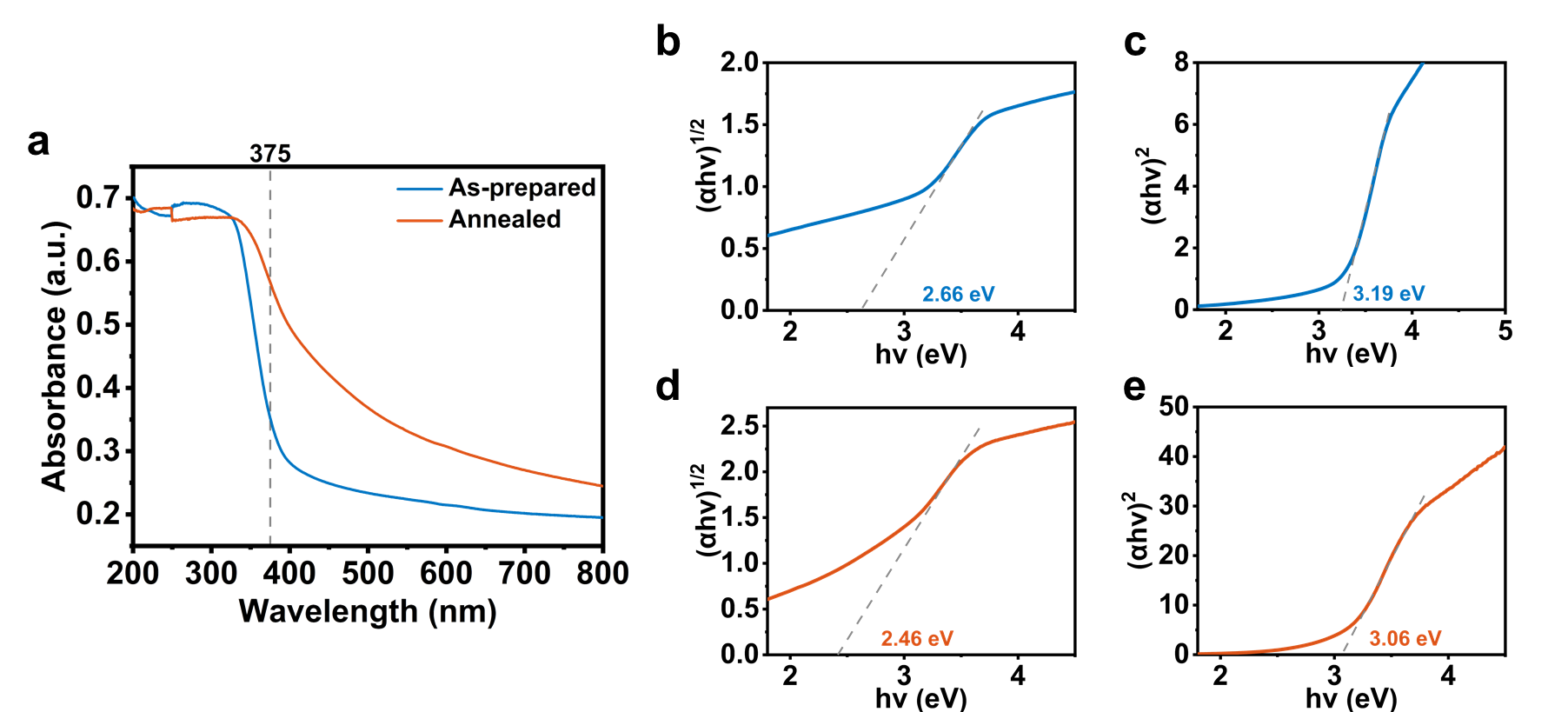


**Figure S21. a,** UV-vis absorption spectra of stripped PTO films from La_0.7_Sr_0.3_MnO_3_/STO substrate. **b,** Tauc indirect band gap of as-prepared film. **c,** Tauc direct band gap of as-prepared film. **d,** Tauc indirect band gap of annealed film. **e,** Tauc direct band gap of annealed film.


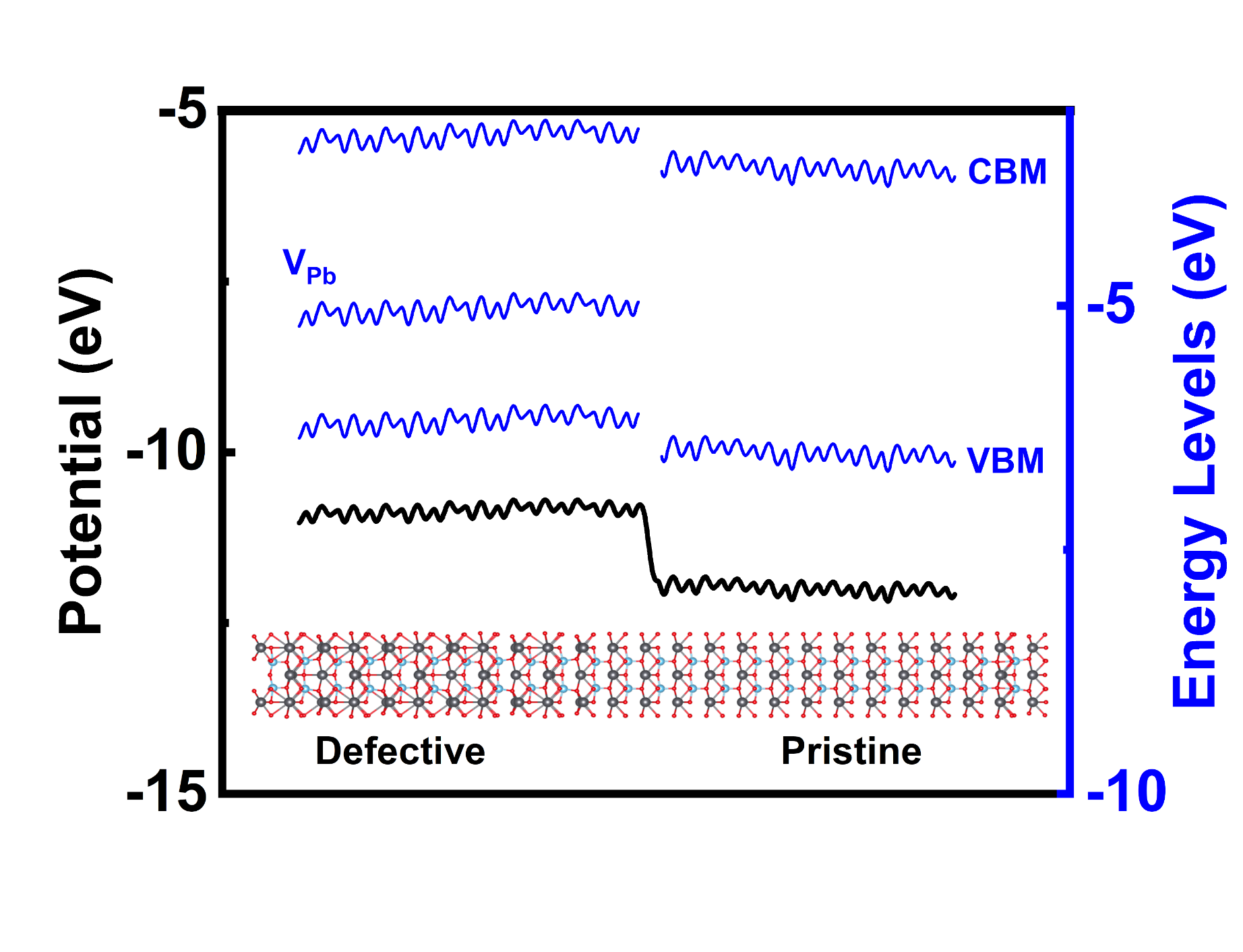


**Figure S22.** Electrostatic potential and energy level position near the cross-over from pristine PTO to defective PTO.


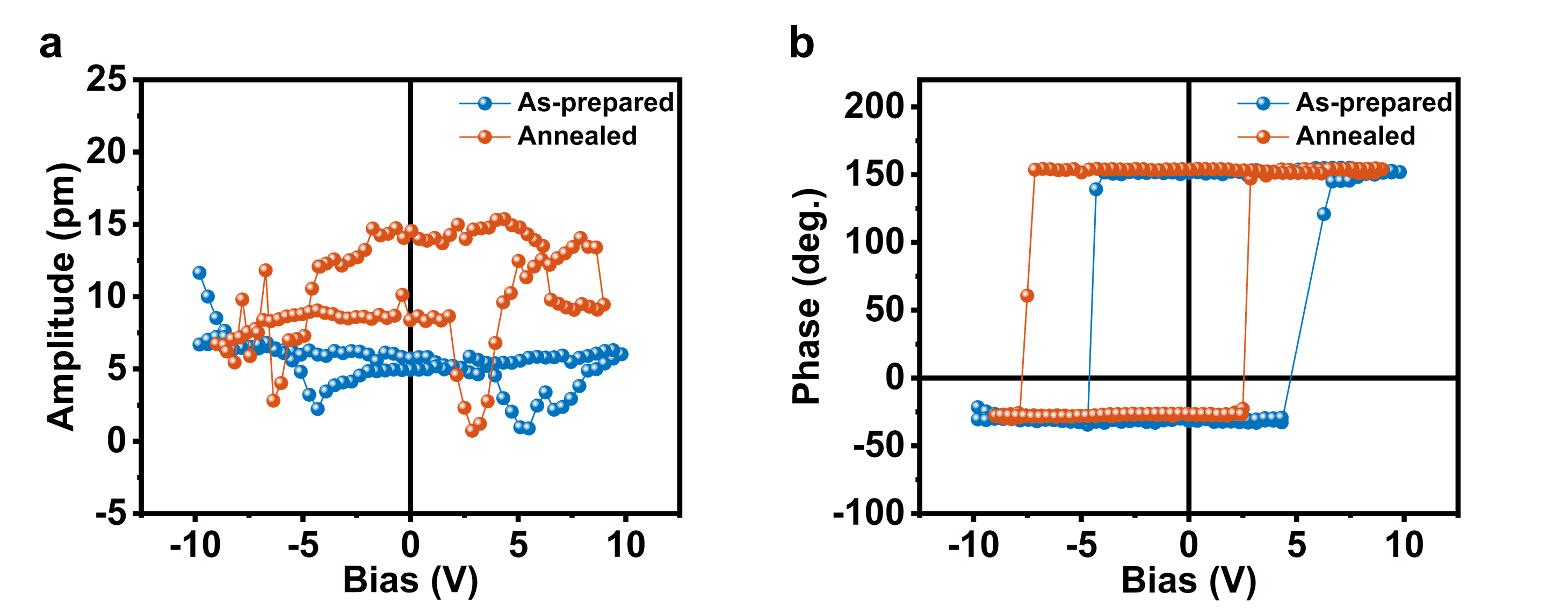


**Figure S23. a,** Piezoresponse amplitude curve with a “butterfly-shape” loop of as-prepared and annealed PTO films. **b,** Piezoresponse phase curve with a hysteresis loop of as-prepared and annealed PTO films.


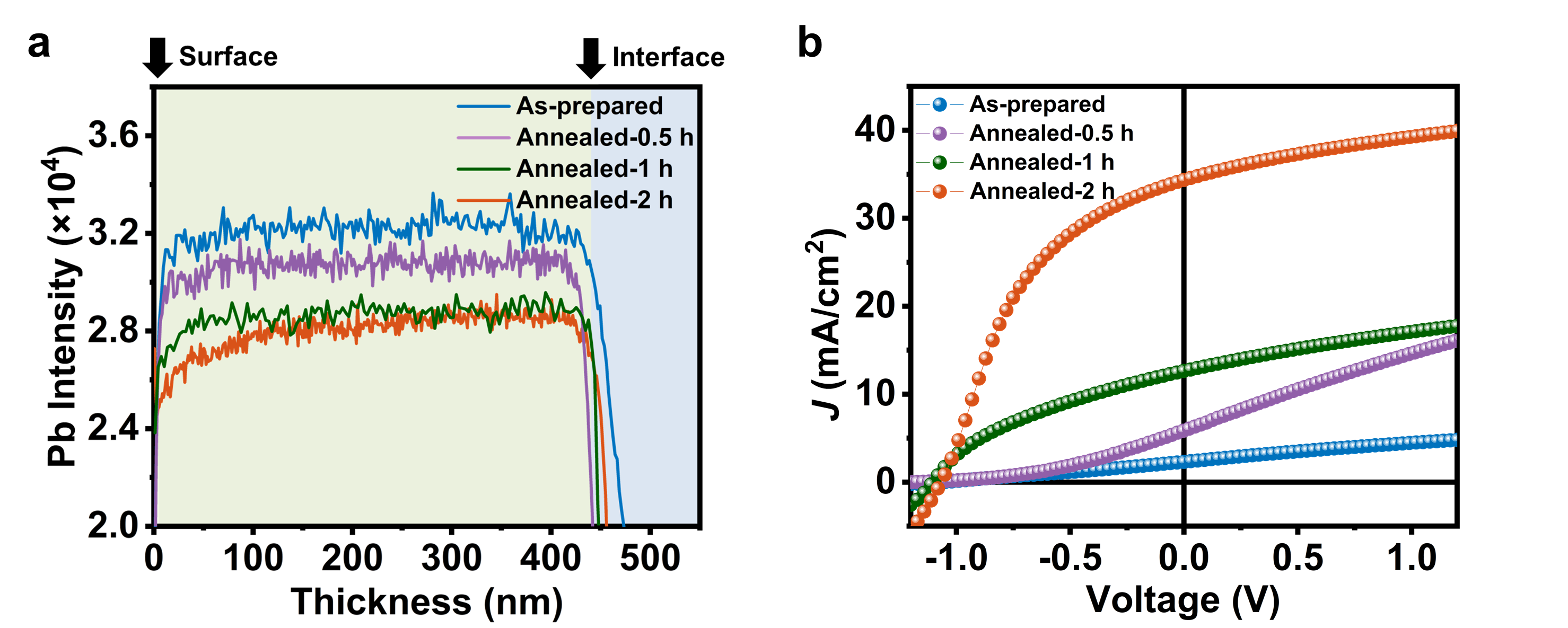


**Figure S24. a,** TOF-SIMS spectra of as-prepared and annealed films at 750℃ with different annealing time (0.5 h, 1 h and 2 h) for Pb element. **b,** *J-V* curves of as-prepared and annealed films at 750℃ with different annealing time (0.5 h, 1 h and 2 h) under the illumination of 500 mW/cm^2^ *I*_light_ from a 375 nm laser.


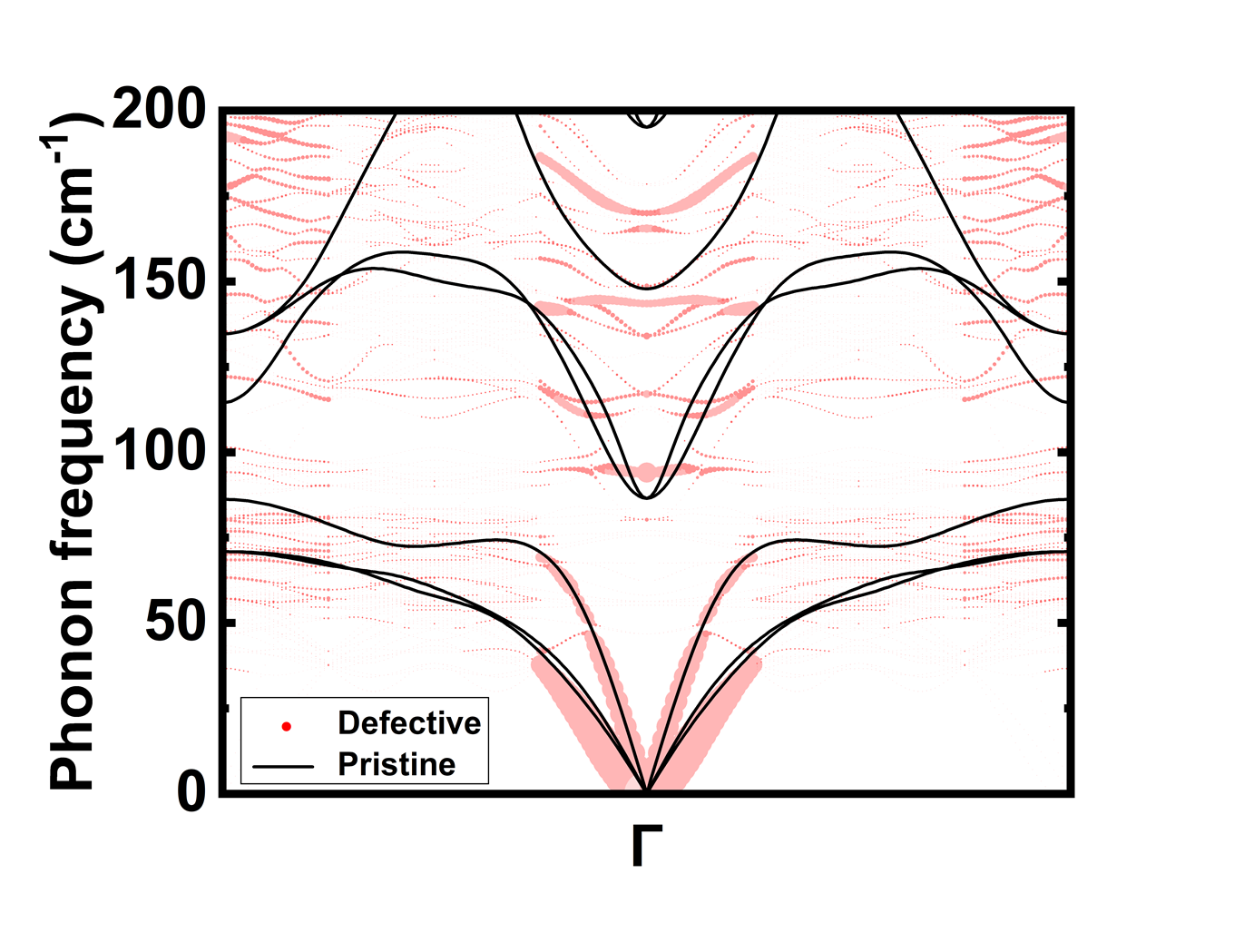


**Figure S25.** The phonon dispersion of defective PTO (red points) and the pristine PTO (black lines). The lowest optical phonon is slightly hardened to a higher frequency, while the acoustic phonons is not significantly changed. The fitted phonon energy (0.13 meV) corresponds to acoustic phonons around 1 cm^-1^, suggesting an acoustic-phonon-dominated process.


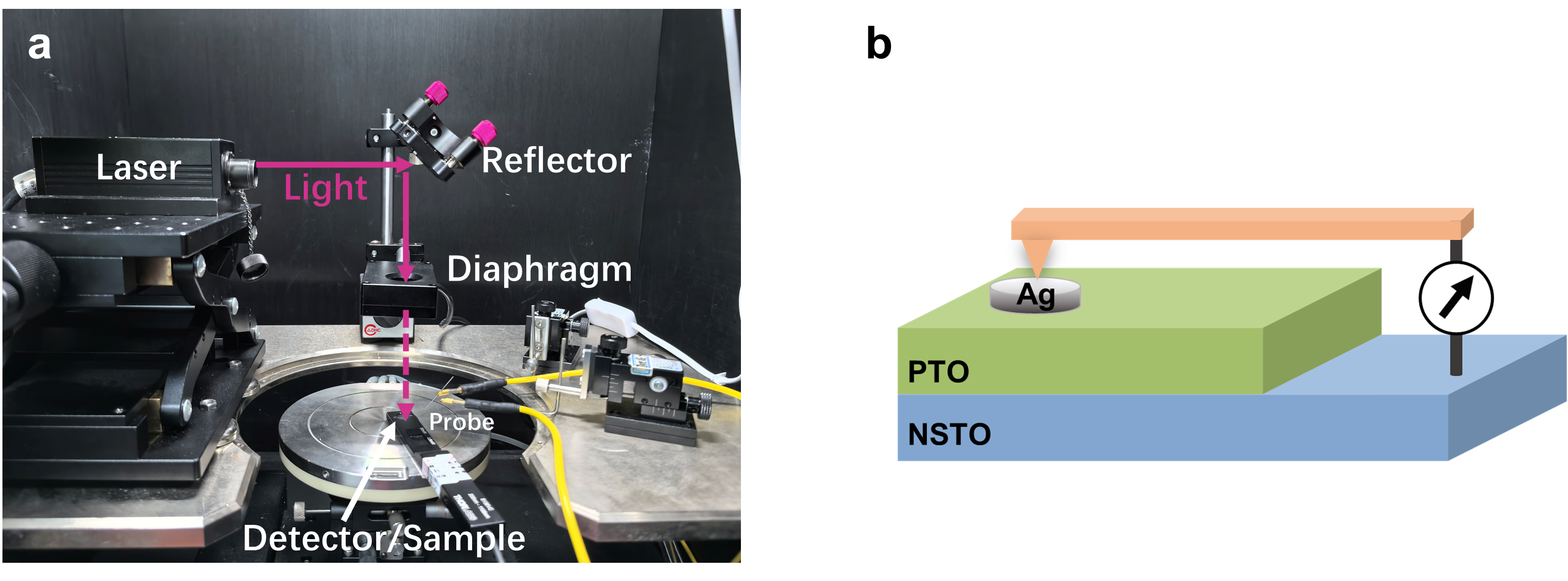


**Figure S26. a,** Digital photo of PV performance test device. **b,** Sketch of the setup for PV measurements.


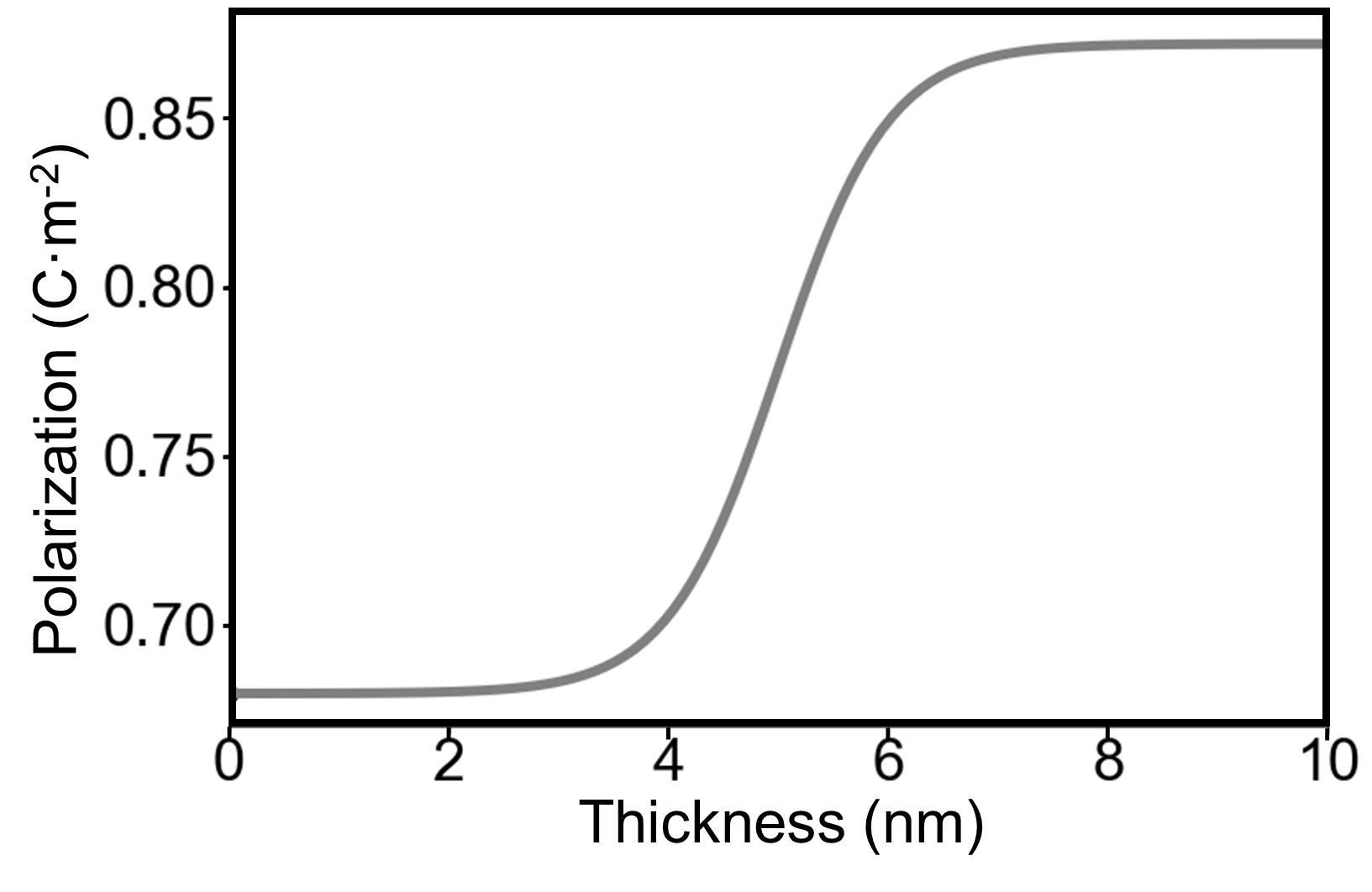


**Figure S27.** Possible polarization profile near the annealed PTO/NSTO interface. The end points are computed from the Berry-phase polarizations, and the profile assumes a hyperbolic tangent function.


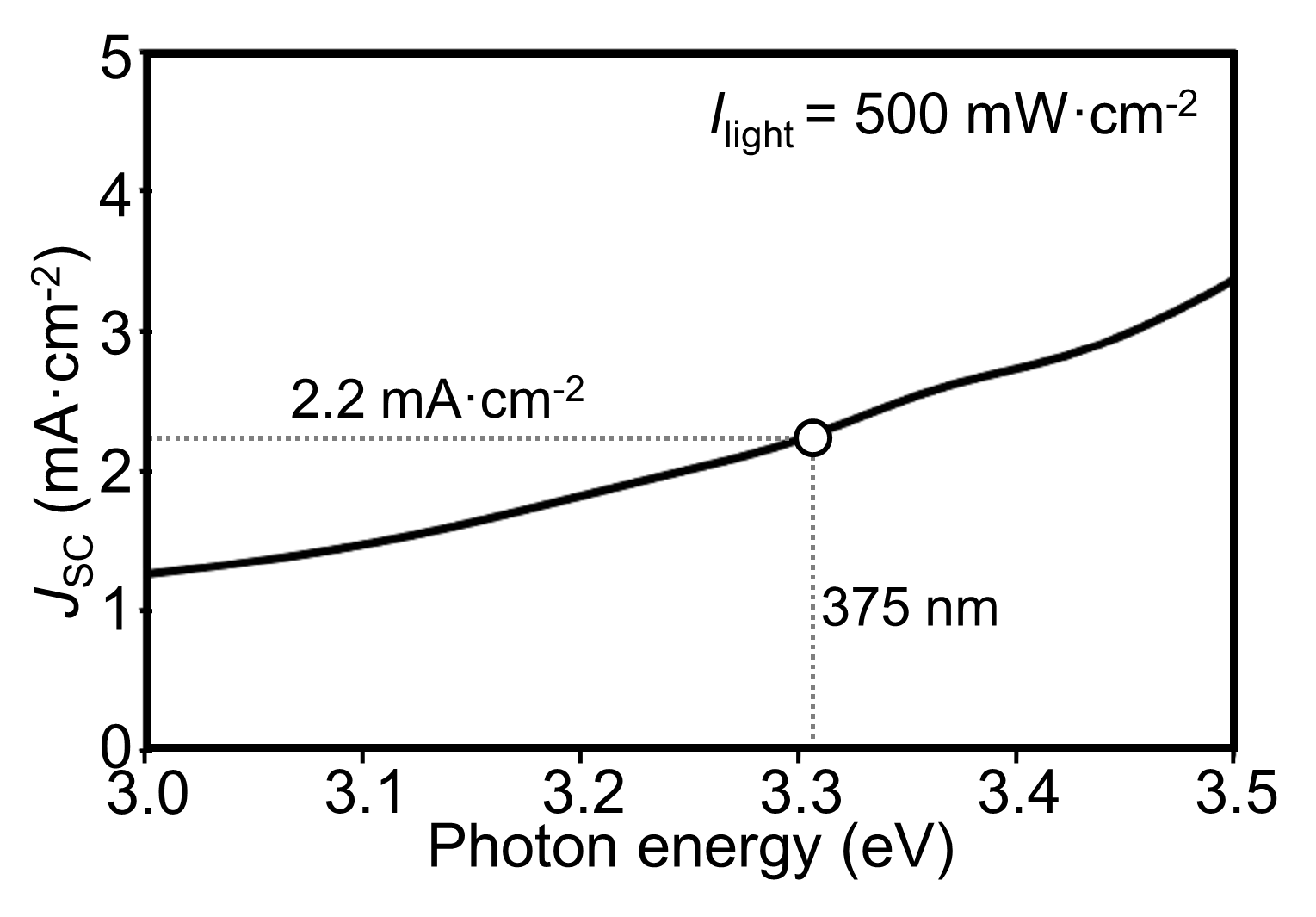


**Figure S28.** Theoretically calculated photocurrent from traditional PV effect for a perfect PTO.

**Table S1.** Lattice parameter and Ti ion displacement information of annealed film obtained from Figure S6.

|  | Lattice parameter *a* (Å) | Lattice parameter *c* (Å) | *c/a* | Ti^4+^ displacements along *a*-axis δ*_a_* (Å) | Ti^4+^ displacements along *c*-axis δ*_c_* (Å) |
| --- | --- | --- | --- | --- | --- |
| **surface** | 3.920±0.026 | 4.175±0.047 | 1.065 | 0.095±0.035 | 0.273±0.025 |
| **Δ*d*=306 nm** | 3.922±0.017 | 4.149±0.022 | 1.058 | 0.102±0.027 | 0.254±0.049 |
| **Δ*d*=159 nm** | 3.926±0.009 | 4.130±0.018 | 1.052 | 0.105±0.048 | 0.190±0.044 |
| **interface** | 3.939±0.013 | 4.113±0.011 | 1.044 | 0.113±0.056 | 0.157±0.057 |

**Table S2.** Summary of *J*_SC_ and *V*_OC_ for all tested films under light and dark conditions.

|  |  |  | As-prepared | | Annealed | |
| --- | --- | --- | --- | --- | --- | --- |
| **Wavelength (nm)** | **Light intensity (mW/cm^2^)** | **PV performance** | **Dark** | **Light** | **Dark** | **Light** |
| **375** | **500** | ***J*_SC_ (mA/cm^2^)** | 1.9×10^-4^ | 2.31 | 2.4×10^-4^ | 34.36 |
|  |  | ***V*_OC_ (V)** | 0.01 | 1.06 | 0.01 | 1.07 |
| **266** | **50** | ***J*_SC_ (mA/cm^2^)** | 4.7×10^-4^ | 4.1×10^-3^ | 4.0×10^-4^ | 4.88 |
|  |  | ***V*_OC_ (V)** | 0.01 | 1.04 | 0.02 | 1.02 |
| **405** | **500** | ***J*_SC_ (mA/cm^2^)** | 1.3×10^-4^ | 0.62 | 1.6×10^-4^ | 15.03 |
|  |  | ***V*_OC_ (V)** | 0.01 | 1.05 | 0.01 | 1.07 |
| **532** | **500** | ***J*_SC_ (mA/cm^2^)** | 3.1×10^-4^ | 1.1×10^-3^ | 7.1×10^-4^ | 2.6×10^-3^ |
|  |  | ***V*_OC_ (V)** | 0.02 | 0.51 | 0.03 | 0.52 |
| **660** | **500** | ***J*_SC_ (mA/cm^2^)** | 2.7×10^-4^ | 2.8×10^-4^ | 4.1×10^-4^ | 5.0×10^-4^ |
|  |  | ***V*_OC_ (V)** | 0.02 | 0.36 | 0.02 | 0.45 |

**Table S3.** Summary of the reported photovoltaic properties in ferroelectric materials.

| **Materials (type)-Publication year** | ***J*_SC_ / mA/cm^2^** | ***V*_OC_ / V** | **Light wavelength / nm** | ***I*_light_ / mW/cm^2^** | **Photoresponsivity (*J*_SC_ /*I*_light_) / A/W** | **Ref.** |
| --- | --- | --- | --- | --- | --- | --- |
| **PTO (Epitaxial film)-2025** | 4.88 | 1.02 | 266 | 50 | **9.76×10^-2^** | **This work** |
|  | 34.36 | 1.07 | 375 | 500 | **6.87×10^-2^** |  |
| **PTO (Epitaxial film)-2023** | 2.153 | 1.15 | 375 | 500 | **4.306×10^-3^** | ^[25]^ |
| **Pb(Zr_0.2_Ti_0.8_)O_3_-625°C (Epitaxial film)-2022** | ~0.19 | ~0.75 | 365 | 228 | **8.33×10^-4^** | ^[67]^ |
| **BDA(EA)_2_Pb_3_Br_10_** **(Bulk single crystal)-2022** | 3.23×10^-3^ | 0.2 | 405 | 38 | **8.5×10^-5^** | ^[68]^ |
| **Pb(In_1/2_Nb_1/2_)O_3_-Pb(Mg_1/3_Nb_2/3_)O_3_-PbTiO_3_ _(24/42/34)_ (Ceramics)-2022** | 6.16×10^-6^ | 23 | 405 | 120 | **5×10^-8^** | ^[69]^ |
| **CuInP_2_S_6_ (****Ultrathin vdW materials prepared by the mechanical exfoliation)-2021** | 0.8 | 1.65 | 405 | 300 | **2.67×10^-3^** | ^[34]^ |
| **(SrTiO_3_)_2_/(BaTiO_3_)_2_/(CaTiO_3_)_2_ superlattices (Epitaxial film)-2021** | 0.49 | / | 405 | 20798 | **2.36×10^-5^** | ^[12]^ |
| **(isopentylammonium)_2_(ethylammonium)_2_Pb_3_I_10_ (Bulk single crystal)-2021** | 1.5×10^-3^ | 0.8 | 637 | 127 | **1.18×10^-5^** | ^[70]^ |
| **BiFeO_3_** **(Epitaxial film)-2020** | 2.88×10^-2^ | 0.715 | 1 Sun AM 1.5G | 100 | **2.88×10^-4^** | ^[71]^ |
| **Ag/BiFeO_3_/ITO-66.1°C (Epitaxial film)-2020** | 0.690 | ~0.6 | 365 | 105.2 | **6.56×10^-4^** | ^[72]^ |
| **Fe:KTa_0.41_Nb_0.59_O_3_ (Bulk single crystal)-2020** | ~2.06×10^-4^ | 6 | 405 | ~1111.11 | **~1.85×10^-7^** | ^[73]^ |
| **Ni-doped PLZT (Ceramics)-2020** | 3.67×10^-5^ | 23 | 405 | 100 | **3.67×10^-7^** | ^[74]^ |
| **La-substituted BiFeO_3_ (Epitaxial film)-2018** | ~2.5×10^-3^ | ~0.55 | halogen lamp | 100 | **~2.5×10^-5^** | ^[16]^ |
| **Ba_0.875_(Bi_0.5_Li_0.5_)_0.125_TiO_3_ (Ceramics)-2018** | 6.5×10^-6^ | 16 | Xenon-arc lamp | 100 | **6.5×10^-8^** | ^[75]^ |
| **Mn-doped BiFeO_3_ (Epitaxial film)-2017** | 1.5×10^-2^ | 3.1 | 515 | 2500 | **6.0×10^-6^** | ^[76]^ |
| **BaTiO_3_ (Bulk single crystal)-2017** | 1.36×10^-4^ | ~0.6 | 375 | 805 | **1.69×10^-7^** | ^[77]^ |
| **[PbTiO_3_]_0.65_[BiNi_2/3_Nb_1/3_O_3–δ_]_0.35_ (Ceramics)-2015** | 1.16×10^-4^ | 10 | 400-780 | 200 | **5.8×10^-7^** | ^[78]^ |
| **BaTiO_3_ 20 nm (Epitaxial film)-2014** | 2.2×10^-3^ | 0.6 | 360 | 300 | **7.33×10^-6^** | ^[79]^ |
| **BaTiO_3_ 50 nm (Epitaxial film)-2014** | 3.8×10^-3^ | 0.65 | 360 | 300 | **1.27×10^-5^** | ^[79]^ |
| **[KNbO_3_]_0.9_[BaNi_1/2_Nb_1/2_O_3-δ_]_0.1_ (Ceramics)-2013** | ~4×10^-5^ | 3.5 | 700 | 4 | **9.0×10^-6^** | ^[7]^ |
| **BiFeO_3_ (Bulk single crystal)-2011** | 1×10^-3^ | 13 | 405 | 8000 | **1.25×10^-7^** | ^[13]^ |
| **BiFeO_3_ (Epitaxial film)-2010** | ~2.8×10^-4^ | 0.3 | 435 | 0.75 | **~3.73×10^-4^** | ^[80]^ |
| **BiFeO_3_ (Bulk single crystal)-2009** | 7.35×10^-3^ | ~0.08 | 532 | ~20 | **~3.675×10^-4^** | ^[5]^ |
| **PLZT_(3/52/48)_ (Ceramics)-2004** | 1.7×10^-3^ | 0.8 | near-ultraviolet | 150 | **1.13×10^-5^** | ^[81]^ |

**Table S4.** Comparison of the speed of sound and deformation potentials (DP) for acoustic phonons in pristine PTO and defective PTO with 1/8 Pb vacancy.

|  | Pristine PTO | PTO with 1/8 V_Pb_ | Relative change |
| --- | --- | --- | --- |
| Longitudinal  speed of sound | 4313 m/s | 4031 m/s | -6.5% |
| Transverse  speed of sound | 2454 m/s | 2246 m/s | -8.5% |
| Valence band  DP (ab axes) | 1.89 eV | 2.62 eV | +38.6% |
| Valence band  DP (c axis) | 0.51 eV | 0.71 eV | +39.2% |
| Conduction band  DP (ab axes) | 1.93 eV | 0.58 eV | -70.0% |
| Conduction band  DP (c axis) | 1.29 eV | 1.00 eV | -22.5% |

**References**

53. G. Kresse and J. Furthmüller, “Efficient iterative schemes for *ab initio* total-energy calculations using a plane-wave basis set,” *Physical Review B* 54(1996): 11169–11186.

54. G. Kresse and D. Joubert, “From ultrasoft pseudopotentials to the projector augmented-wave method,” *Physical Review B* 59(1999): 1758–1775.

55. P. E. Blöchl, “Projector augmented-wave method,” *Physical Review B* 50(1994): 17953–17979.

56. J. P. Perdew, K. Burke and M. Ernzerhof, “Generalized gradient approximation made simple,” *Physical Review Letters* 77(1996): 3865–3868.

57. J. Heyd, G. E. Scuseria and M. Ernzerhof, “Erratum: “Hybrid functionals based on a screened Coulomb potential” [J. Chem. Phys. 118, 8207 (2003)],” *Journal of Chemical Physics* 124(2006): 219906.

58. A. V. Krukau, O. A. Vydrov, A. F. Izmaylov and G. E. Scuseria, “Influence of the exchange screening parameter on the performance of screened hybrid functionals,” *Journal of Chemical Physics* 125(2006): 224106.

59. Q. Wu, S. Zhang, H.-F. Song, M. Troyer and A. A. Soluyanov, “WannierTools: An open-source software package for novel topological materials,” *Computer Physics Communications* 224(2018): 405–416.

60. M. L. Cohen and S. G. Louie 2016, “*Fundamentals of Condensed Matter Physics*.” Cambridge University Press, Cambridge. ISBN 9780521513319.

61. R. Fei, L. Z. Tan and A. M. Rappe, “Shift-current bulk photovoltaic effect influenced by quasiparticle and exciton,” *Physical Review B* 101(2020): 045104.

62. P. Giannozzi, O. Andreussi, T. Brumme, et al., “Advanced capabilities for materials modelling with Quantum ESPRESSO,” *Journal of Physics: Condensed Matter* 29(2017): 465901.

63. P. Giannozzi, S. Baroni, N. Bonini, et al., “QUANTUM ESPRESSO: a modular and open-source software project for quantum simulations of materials,” *Journal of Physics: Condensed Matter* 21(2009): 395502.

64. H. Lee, S. Poncé, K. Bushick, et al., “Electron-phonon physics from first principles using the EPW code,” *npj Computational Materials* 9(2023): 156.

65. R. Resta and D. Vanderbilt 2007, “*Theory of Polarization: A Modern Approach. in Physics of Ferroelectrics: A Modern Perspective.*” Springer, Berlin, Heidelberg. ISBN 978-3-540-34590-9.

66. B. Meyer and D. Vanderbilt, “Ab initio study of ferroelectric domain walls in PbTiO_3_,” *Physical Review B* 65(2002): 104111.

67. Q. Huang, Z. Fan, J. Rao, et al., “Significant modulation of ferroelectric photovoltaic behavior by a giant macroscopic flexoelectric effect induced by strain‐relaxed epitaxy,” *Advanced Electronic Materials* 8(2022): 2100612.

68. H. Ye, Y. Peng, X. Shang, et al., “Self‐powered visible‐infrared polarization photodetection driven by ferroelectric photovoltaic effect in a Dion-Jacobson hybrid perovskite,” *Advanced Functional Materials* 32(2022): 2200223.

69. X. Qi, K. Li, E. Sun, et al., “Large photovoltaic effect with ultrahigh open-circuit voltage in relaxor-based ferroelectric Pb(In_1/2_Nb_1/2_)O_3_-Pb(Mg_1/3_Nb_2/3_)O_3_-PbTiO_3_ ceramics,” *Journal of Materials Science & Technology* 104(2022): 119–126.

70. S. Han, M. Li, Y. Liu, et al., “Tailoring of a visible-light-absorbing biaxial ferroelectric towards broadband self-driven photodetection,” *Nature Communications* 12(2021): 284.

71. M. Wang, H. Wei, Y. Wu, et al., “Polarization-enhanced bulk photovoltaic effect of BiFeO_3_ epitaxial film under standard solar illumination,” *Physics Letters A* 384(2020): 126831.

72. R. Zhao, N. Ma, K. Song and Y. Yang, “Boosting photocurrent via heating BiFeO_3_ materials for enhanced self‐powered UV photodetectors,” *Advanced Functional Materials* 30(2020): 1906232.

73. F. Huang, C. Hu, Z. Xian, et al., “Photovoltaic properties in an orthorhombic Fe doped KTN single crystal,” *Optics Express* 28(2020): 34754.

74. G. Huangfu, H. Xiao, L. Guan, et al., “Visible or Near-infrared light self-powered photodetectors based on transparent ferroelectric ceramics,” *ACS Applied Materials & Interfaces* 12(2020): 33950–33959.

75. S. Pal, A. B. Swain, P. P. Biswas, et al., “Giant photovoltaic response in band engineered ferroelectric perovskite,” *Scientific Reports* 8(2018): 8005.

76. H. Matsuo, Y. Noguchi and M. Miyayama, “Gap-state engineering of visible-light-active ferroelectrics for photovoltaic applications,” *Nature Communications* 8(2017): 207.

77. J. Li, C. Ge, K. Jin, et al., “Self-driven visible-blind photodetector based on ferroelectric perovskite oxides,” *Applied Physics Letters* 110(2017): 142901.

78. H. Liu, J. Chen, Y. Ren, et al., “Large photovoltage and controllable photovoltaic effect in PbTiO_3_‐Bi(Ni_2/3+x_Nb_1/3-x_)O_3–δ_ ferroelectrics,” *Advanced Electronic Materials* 1(2015): 1400051.

79. A. Zenkevich, Yu. Matveyev, K. Maksimova, R. Gaynutdinov, A. Tolstikhina and V. Fridkin, “Giant bulk photovoltaic effect in thin ferroelectric BaTiO_3_ films,” *Physical Review B* 90(2014): 161409.

80. W. Ji, K. Yao and Y. C. Liang, “Bulk photovoltaic effect at visible wavelength in epitaxial ferroelectric BiFeO_3_ thin films,” *Advanced Materials* 22(2010): 1763–1766.

81. M. Ichiki, R. Maeda, Y. Morikawa, Y. Mabune, T. Nakada and K. Nonaka, “Photovoltaic effect of lead lanthanum zirconate titanate in a layered film structure design,” *Applied Physics Letters* 84(2004): 395–397.
